# MetaGen: reference-free learning with multiple metagenomic samples

**DOI:** 10.1186/s13059-017-1323-y

**Published:** 2017-10-03

**Authors:** Xin Xing, Jun S. Liu, Wenxuan Zhong

**Affiliations:** 1 Department of Statistics, University of Georgia, Athens, 30602 GA USA; 2 Department of Statistics, Harvard University, Cambridge, 02138 MA USA; 3 Center for Statistical Science & Department of Industry Entering, Beijing, 100084 China

**Keywords:** Metagenomics, Binning, Mixture model, Multinomial, Unsupervised learning

## Abstract

A major goal of metagenomics is to identify and study the entire collection of microbial species in a set of targeted samples. We describe a statistical metagenomic algorithm that simultaneously identifies microbial species and estimates their abundances without using reference genomes. As a trade-off, we require multiple metagenomic samples, usually ≥10 samples, to get highly accurate binning results. Compared to reference-free methods based primarily on *k*-mer distributions or coverage information, the proposed approach achieves a higher species binning accuracy and is particularly powerful when sequencing coverage is low. We demonstrated the performance of this new method through both simulation and real metagenomic studies. The MetaGen software is available at https://github.com/BioAlgs/MetaGen.

**Electronic supplementary material:**

The online version of this article (doi:10.1186/s13059-017-1323-y) contains supplementary material, which is available to authorized users.

## Background

Due to the rapid advances of high-throughput sequencing technologies, metagenomics, which investigates the genetic contents of the entire collection of microbial species in a set of environmental samples, is becoming a major tool for studying microbial ecology, evolution, and diversity, as well as linking microbial features to the surrounding environment or human health [1–3].

In the past decade, many methods have been proposed for estimating microbial compositions from metagenomic sequencing data, with a majority focused on targeted sequencing data that provide information only on a few selected genes, such as the 16S rRNA gene [4]. Because the targeted approach requires the sequencing of only a limited number of genes instead of hundreds of microbial genomes, it is cheap and computationally efficient. The trade-offs are that it can reach only a fairly high taxonomic rank, i.e., it has a relatively low resolution in differentiating distinct species, and that it cannot provide information regarding other important genomic components. Moreover, statistical estimation based on targeted sequencing data can be biased because the polymerase chain reaction primers used for amplifying the targeted genes, such as the 16S rRNA gene, have different levels of sensitivity in different species [5].

Because of the drastic cost reduction in next-generation sequencing technologies and the disadvantages of targeted-gene-based approaches, genome-wide shotgun sequencing has become the dominant technique in metagenomic studies. The genomic fragments obtained from metagenomic samples are binned into different species or taxonomical bins either according to the fragments’ similarities to some known reference genomes or according to the sequence composition similarities (e.g., similarities between *k*-mer distributions [6] or oligonucleotide frequencies [7]). This class of approaches is referred to as binning methods. Reference-based binning methods such as MEGAN [8], MetaPhyler [9], Kraken [10], and CLARK [11] require us to know the reference genomes of the interested microbial species, which can be a serious limitation. In contrast, the *k*-mer or the oligonucleotide-frequency-based methods are reference-free. However, the binning accuracy of *k*-mer-based method can be significantly compromised because the *k*-mer distributions estimated from short contigs (e.g., <10 kb) can be far from their corresponding whole-genome *k*-mer distributions. Meanwhile, the effectiveness of *k*-mer-based methods is also diminished when the microbial community under consideration contains organisms with moderate to high sequence similarities. To improve the *k*-mer-based approaches, coverage-based methods such as CONCOCT [12], MaxBin [13], MetaBAT [14], Groopm [15], and VizBin [16], have been developed to integrate the coverage information (i.e., the average number of short reads covering each base pair of a contig after alignment) with the sequence composition information. Although integrating coverage information can significantly improve the binning accuracy, how to balance the *k*-mer information with the coverage information is by no means a banal development. Our simulation studies suggest that most of the existing coverage-based methods still fail in distinguishing genetically similar species. Moreover, the coverage estimate is biased when a species does not have adequate coverage or when the sequencing bias is high.

In this article, we propose a reference-free and distribution-free binning method, MetaGen, which makes use of the relative abundance information from multiple samples to cluster contigs into different species bins and relies on the Bayesian information criterion (BIC) to determine the number of species in the samples. Since MetaGen uses solely the cross-sample abundance patterns for binning, we recommend that the number of samples in consideration should be larger than ten. Compared to existing unsupervised binning methods, MetaGen not only clusters short contigs accurately for samples with low coverage but also has the ability to distinguish species with high sequence similarities. In addition, MetaGen can estimate the relative abundance of cultured and uncultured species simultaneously, which provides a way to study distributional changes in microbial colonies dynamically and spatially. Moreover, MetaGen is not susceptible to sequencing biases, which is an important advantage compared with many existing methods. MetaGen is computationally efficient and can easily handle large data sets with more than 500,000 contigs.

## Results

### Multi-sample reference-free binning: an overview

We consider metagenomic sequencing data consisting of short reads from the genomes of the organisms in the samples. The first step in almost all analysis methods is to connect overlapping short reads from the pooled sample into longer sequences, termed contigs. The *k*-mer-based reference-free methods proceed to bin (i.e., cluster) these contigs, regarding them as coming from the same or similar species, according to similarities among the *k*-mer distributions of these contigs. Our proposed method, MetaGen, however, uses the relative abundance information of the contigs across multiple samples to cluster them. Thus, whereas the *k*-mer-based methods need to assume that contigs derived from the same species have similar *k*-mer distributions, MetaGen assumes that abundances of different species vary across multiple samples.

Since each contig is composed of many short reads from all samples, we define each contig’s *sample profile* as the vector of percentages of short reads mapped from different samples. As the genome of a species can be thought of as the longest possible contig, we refer to the similarly defined short-read percentage vector as the species’ sample profile. In theory, a contig’s sample profile should be the same as the sample profile of the species that contains the contig (if we assume that the contig is long enough for a unique mapping). Thus, if two contigs have similar sample profiles, they are likely derived from the same genome. MetaGen models the mapped short-read counts of each contig by a mixture of multinomial distributions, with each of its mixture components representing a distinct species. The limitation of MetaGen is that if two species have nearly proportional abundances in all the samples, their corresponding contigs will tend to have highly correlated sample profiles, which makes it difficult for MetaGen to differentiate the two species. As shown by our simulation studies, however, this difficulty can be alleviated by increasing the sequencing depth.

### Statistical deconvolution of metagenomic samples

**Fig. 1:**
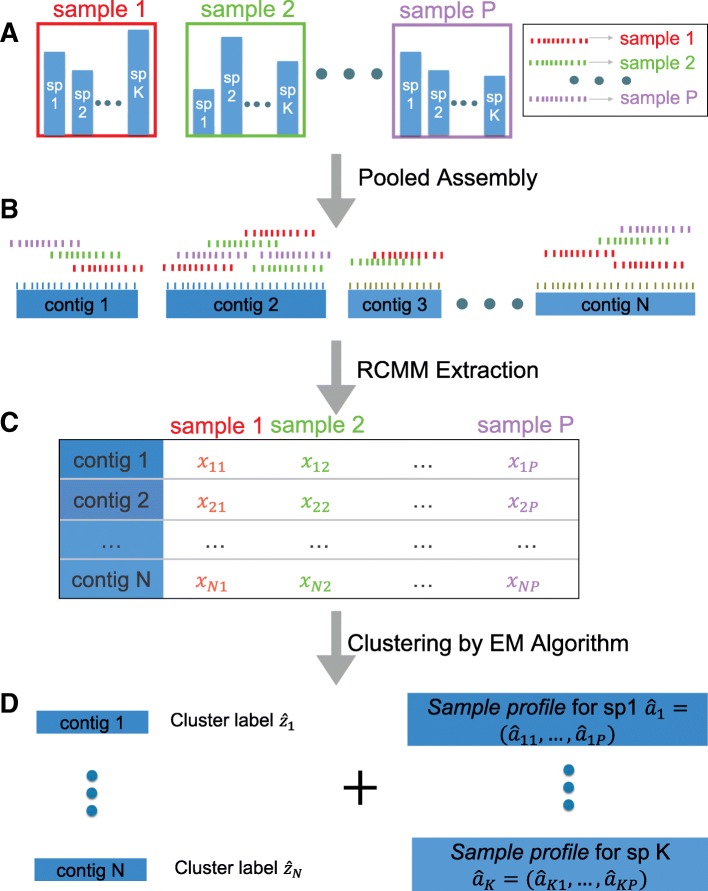
MetaGen pipeline. **a** Sequencing the DNA of *P* metagenomic samples. **b** Pooled assembly for multiple samples. **c** Constructing the RCMM. **d** Clustering the contigs and estimating the sample profile by the EM algorithm. EM expectation-maximization, RCMM read counts mapping matrix

As explained previously, if two contigs have very similar sample profiles, they are likely part of the same species’ genome. Let us assume that *N* contigs were obtained from *P* metagenomic samples, with a total of *K* species involved. The extracted *read counts mapping matrix* (RCMM) has *N* rows and *P* columns, with its (*i*,*j*)th entry recording the read count from the *j*th sample mapped on to the *i*th contig, as shown in step C of Fig. 1. Thus, each row of RCMM is proportional to the sample profile of a contig. A direct clustering of the rows of RCMM provides information about the number of species and their distributions in the samples.

Let **X**_*i*_, *i*=1,…,*N*, denote the row vectors of the RCMM, each of length *P*, and let *Z*_*i*_ take values in {1,…,*K*}, indicating from which species contig *i* is derived. We assume that the *Z*_*i*_’s are independent, and *P*(*Z*_*i*_=*k*)=*π*_*k*_, with the probability vector ***π***=(*π*_1_,…,*π*_*K*_). Furthermore, we assume that given the species label *Z*_*i*_, **X**_*i*_ follows a multinomial distribution:

1 $$ \Pr(\mathbf{X}_{i} = \mathbf{x}_{i}|Z_{i}= k) = \frac{n_{i}}{x_{i1}!\dots x_{iP}!} a_{k 1}^{x_{i1}} \dots a_{k P}^{x_{iP}},  $$

where **a**_*k*_=(*a*_*k*1_,…,*a*_*kP*_), ${\sum \nolimits }_{j=1}^{P} a_{kj}=1$, is the sample profile of the *k*th species, and $n_{i}={\sum \nolimits }_{j=1}^{P}x_{ij}$ is the total number of mapped reads on the *i*th contig. Let *A* denote the *K*×*P* sample profile matrix constructed by stacking up the **a**_*k*_’s, and let *θ*=(***π***,*A*). Treating *Z*_*i*_ as missing data, we have the complete-data likelihood function as

2 $$\begin{array}{*{20}l} {}L(\theta; \mathbf{x}_{1},&\dots,\mathbf{x}_{N},z_{1},\dots,z_{N})\\ &=\prod_{i=1}^{N}\sum\limits_{k=1}^{K}\pi_{k} \mathbf{1}(z_{i}=k)\frac{n_{i}}{x_{i1}!\dots x_{iP}!} a_{k 1}^{x_{i1}}\dots a_{k P}^{x_{iP}}, \end{array} $$

where **1**(·) is an indicator function. The maximum likelihood estimate of *θ* can be obtained by the expectation-maximization (EM) algorithm [17], which iterates the following two steps:

**E-step**: Calculate *Q*(*θ*|*θ* ^(*t*)^), the expectation of the complete-data log-likelihood function based on the parameter fixed at *θ* ^(*t*)^:

3 $$ \begin{aligned} Q\left(\theta\mid\theta^{(t)}\right) & = \sum\limits_{i=1}^{N} \sum\limits_{k=1}^{K} \hat{q}_{ik}^{(t)}\left[\log\pi_{k}+\sum\limits_{j=1}^{P}x_{ij}\log(a_{kj}) \right], \end{aligned}  $$

where

$$\hat{q}_{ik}^{(t)} = \frac{ {\pi_{k}^{(t)} {a_{k 1}^{(t)}}^{x_{i1}} \dots {a_{k P}^{(t)}}^{x_{iP}}}} {\left[{\sum\nolimits}_{l=1}^{K}\pi_{l}^{(t)} {a_{l 1}^{(t)}}^{x_{i1}} \dots {a_{l P}^{(t)}}^{x_{iP}}\right]}. $$

**M-step**: Find $\hat {\theta }$ that maximizes the function *Q*(*θ*|*θ* ^(*t*)^). This leads to

4 $$ \pi_{k}^{(t+1)} \propto \sum\limits_{i=1}^{N} \hat{q}_{ik}^{(t)} \ \ \text{and} \ \ a_{kj}^{(t+1)}\propto \sum\limits_{i=1}^{N} \hat{q}_{ik}^{(t)} x_{ij}.  $$

#### Initialization and final clustering

Although each EM iteration increases the observed-data likelihood function, the algorithm is not guaranteed to converge to the global maximum. We, thus, employed the following initialization strategy. We first select the 10–30% contigs with the largest number of mapped reads and cluster the selected contigs into *K* species using hierarchical clustering with their pairwise distance defined by

$$d(\mathbf{x}_{1},\mathbf{x}_{2}) = 1 - \frac{{\sum\nolimits}_{j=1}^{P}x_{1 j}x_{2 j}}{\sqrt{{\sum\nolimits}_{j=1}^{P}x_{1 j}^{2}{\sum\nolimits}_{j=1}^{P}x_{1 j}^{2}}}. $$

The class mean of species *k* is then used as the starting values $a^{(0)}_{kj}$, *j*=1,…,*P*. With the maximum likelihood estimate $\hat {\theta }$ obtained by the EM algorithm, we assign each **x**_*i*_ to the species with the highest posterior probability, i.e., we set $\hat {z}_{i} = \arg \!\min _{k} \hat {q}_{ik} $, *i*=1,…,*N*.

### Determining the number of species in the samples

Since the number of species is generally unknown in most applications, we employed BIC [18, 19] to select the number of species. The BIC score for our model with *K* species is defined as

5 $$ \text{BIC}(K) = -2\log L(\hat{\theta}; \mathbf{x}_{1},\dots,\mathbf{x}_{N}) + (KP + K)\log(N).  $$

We determine the number of species $\tilde {K}$ by minimizing this score, i.e.,

6 $$ \tilde{K} = \arg\!\min_{K} \text{BIC}(K).   $$

In practice, we gradually increase the number of species and stop when the BIC score begins to increase. Our simulation studies showed that the criterion worked satisfactorily in accurately determining the number of species in the studies.

### Comparison with coverage-based metagenomic binning methods

There are two types of information contained in metagenomic data: the sequence content information and the sequence quantity information (i.e., the numbers of mapped reads of constructed contigs). The sequence content information has been extensively used in existing metagenomic binning methods, whereas the sequence quantity information is much less used. A few exceptions (such as CONCOCT, MaxBin, and MetaBAT) bin contigs together if their sequencing coverages (the average number of reads that can be aligned to a reference base) are similar. These methods intrinsically assume that no fragment of any involved genome in the sample has positional bias. They work well for GC-neutral or GC-rich species, in which the regional GC bias is not a serious issue. As shown in [20–22], however, the sequencing coverage can be highly variable along the genome, especially for species with a low GC content. For example, it was shown in [21] that *Beta vulgaris BAC ZR-47B15* has nearly 7 times more coverage in GC-rich regions than in GC-poor regions. Consequently, binning contigs based on their coverage similarities is highly susceptible to sequencing bias. In contrast, MetaGen is less susceptible to sequencing bias since it bins contigs based on the ratio of the mapped-read counts (i.e., the sample profile). Sequencing biases do not affect the sample profile because these biases are the same across samples and, thus, can be canceled out. In other words, two contigs from the same species can still be binned together even if their observed coverage is very different due to positional biases.

Another unique feature of MetaGen is that it does not use the sequence (content) information in binning, because the information gain is offset by undesirable sequencing biases and high computational costs, especially when there are short contigs produced from data with relatively low sequencing coverages. As reviewed previously, short contigs are more susceptible to positional and sequencing biases. As shown in our simulation studies, for contigs shorter than 5000 bps, including the sequence information did not increase the binning accuracy, but greatly increased the computational complexity. Another reason for not using the sequence information in MetaGen is that features summarized from the sequence information and those from sequencing coverages are usually at different scales. An ad hoc combination of the two types of information can make the computation unstable, since one type may completely dominate the other. A potential remedy is to weigh the sequence features and sequencing coverage information properly so that the contribution from each source is on the same scale [14]. However, choosing a data-driven weight significantly increases the computational burden without bringing much improvement, most of the time.

Finally, MetaGen directly models short-read counts rather than their transformations as proposed in some recent papers. Thus, it does not need to add deliberately a small pseudo-count to zero coverage values when calculating their logarithmic transformations as suggested in CONCOCT. Moreover, MetaGen avoids using inappropriate Gaussian distributions for non-negative zero-inflated observations as in MetaBAT, which can be important especially for low-coverage data.

### Simulation studies

To investigate how the binning accuracy was affected by other parameters, such as the sequencing depth, the sample size (the number of samples), and the number of species, we conducted extensive simulations to compare MetaGen with three state-of-the-art reference-free binning methods: CONCOCT [12], MaxBin [13], and MetaBAT [14], and one reference-based method, CLARK [11]. The names of the species (or strains) used for all the setups are given in Additional file 1: Tables S1–S3. All the algorithms compared here were implemented on a computer configured with 2× Intel Xeon E5-2670 and 8× 32 GB RAM. Under all the simulation setups, MetaGen is at least 10 times faster than other reference-free binning methods (Additional file 1: Figure S1).

#### How binning accuracy is affected by sequencing depth

**Fig. 2:**
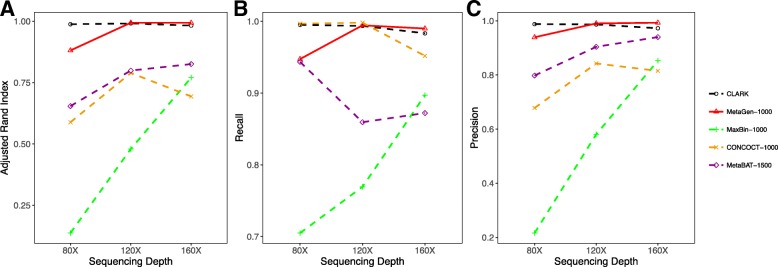
**a** Adjusted rand index, **b** recall, and **c** precision of CLARK, MetaGen, MaxBin, CONCOCT, and MetaBAT evaluated under different sequencing depths for 80 samples and 100 species

First, we examined three sequencing depths for the pooled sample: 80× (1× per sample), 120× (1.5× per sample), and 160× (2× per sample). Short reads from 100 species mixed in a randomly generated proportional distribution were independently simulated for each of the 80 samples. Because all the methods except MetaGen can be significantly impaired for contigs shorter than 1000 bps, we used only the subset of contigs with a length longer than 1000 bps for CONCOCT, MetaGen, MaxBin, and CLARK. For MetaBAT, we used contigs longer than 1500 bps, which is the default minimum length for contigs that can be used in MetaBAT. As shown in Fig. 2, MetaGen performed well at all sequence depths by all three measures: precision, recall, and the adjusted rand index (ARI; a combination of the precision and recall measurements), especially for data with very low sequencing depth. For example, for 1× per sample, MetaGen achieved ARI of 0.88, whereas CONCOCT, MaxBin, and MetaBat had ARI of only 0.59, 0.14, and 0.66, respectively.

It is clear that CLARK outperformed almost all the reference-free methods, especially when the sequence depth is low, because we give a significant advantage to CLARK by assuming that all the reference genomes are known (unrealistic, though). It is also shown in Fig. 2 that the benefit of knowing the reference genome is not so significant when the sequence depth is high enough (say, 1.5× per sample). In fact, the binning accuracy for CLARK is worse than for MetaGen by a tiny margin at 2× per sample due to the alignment error generated by quickly approximating the similarities between contigs and the reference genomes using CLARK. The accuracy of reference-based binning methods can be improved by using BLAST, but the computational cost would be intolerably high.

#### How binning accuracy is affected by sample size

**Fig. 3:**
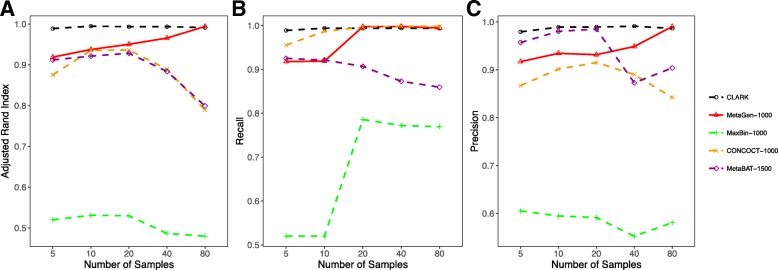
**a** Adjusted rand index, **b** recall, and **c** precision of CLARK, MetaGen, MaxBin, CONCOCT, and MetaBAT evaluated under different numbers of samples for 120× sequencing depth and 100 species

In this experiment, we let the sample size vary from 5 to 80 for 100 species with the pooled sequencing depth at 120×. We followed the same rule as used in the first experiment to generate each metagenomic sample and select subsets of contigs. Note that the per sample sequencing depth in this experiment decreased as we increased the sample size. Since the pooled sequencing depth was fixed, a contig’s coverage in a single sample decreased with the increase in the sample size. As shown in Fig. 3, the binning accuracy decreased for all the existing coverage-based binning methods because the approximate distribution of the log-transformation of the sequencing coverage, which was used to bin contigs, performs badly if the per sample coverage is low (near zero, for example).

However, increasing the sample size is a blessing for MetaGen, as the larger the sample size, the higher the discrimination power of the ratio and the higher the binning specificity. As shown in our simulation studies, the precision increased from 0.93 to 0.99 as we increased the sample size, which in turn led to the increases in ARI.

#### How binning accuracy is affected by number of species

Here we increased the number of species from 50 to 100 and 150, with the pooled sequencing depth fixed at 120× and the sample size fixed at 80. Again, due to the fixed pooled sequencing depth, contigs tend to be shorter for a larger number of species. Thus, increasing the number of species can lead to a higher binning error rate for all methods except MetaGen, because all other methods use *k*-mer distribution similarities for binning and consequently suffer from high binning errors, especially for contigs from genetically similar species.

**Fig. 4:**
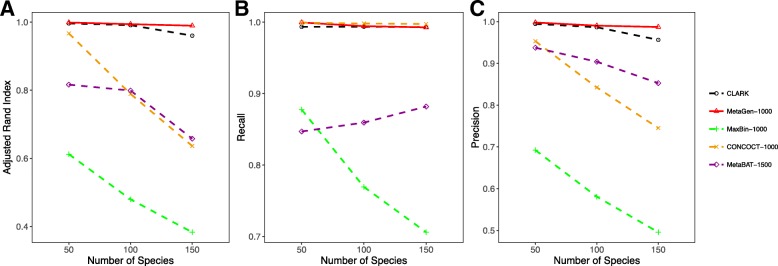
**a** Adjusted rand index, **b** recall, and **c** precision of CLARK, MetaGen, MaxBin, CONCOCT, and MetaBAT evaluated under different numbers of species for 120× sequencing depth and 80 samples

Compared to all the methods that use sequencing information, MetaGen uses only the abundance variation across samples and is consequently less susceptible to the lengths of contigs and more robust for data with a large number of species. As illustrated in Fig. 4, the binning accuracy of MetaGen did not change significantly as we increased the number of species.

#### How binning accuracy is affected by sequence similarity

**Fig. 5:**
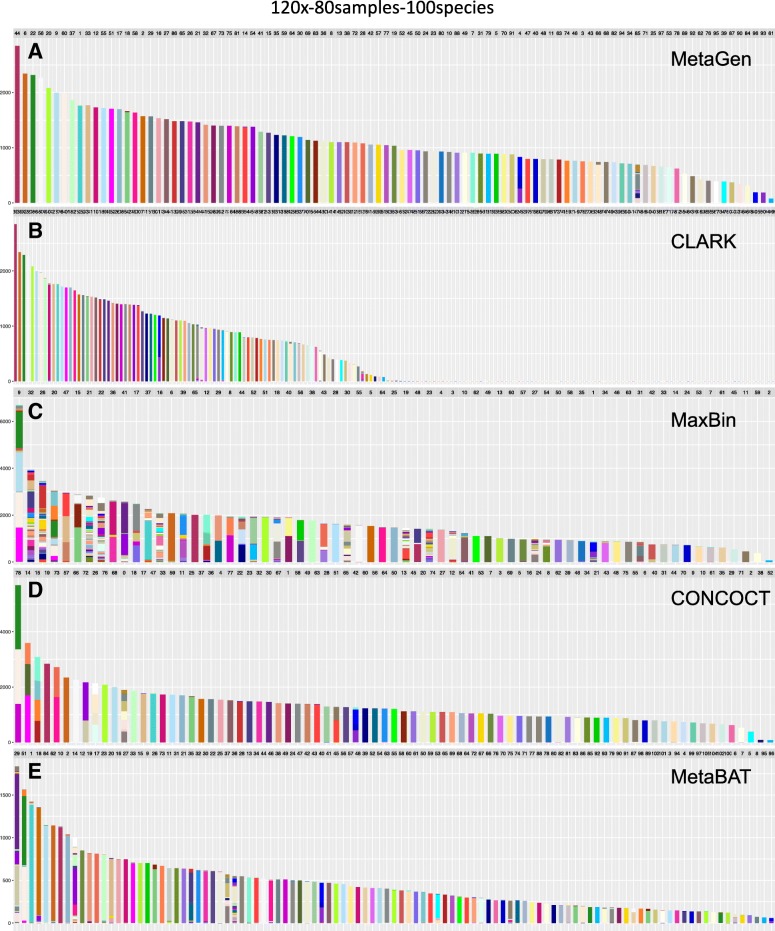
Binning results. For **a** MetaGen, **b** CLARK, **c** MaxBin, **d** CONCOCT, and **e** MetaBAT for 120× sequencing depth, 80 samples, and 100 species (represented by different colors). Each bar represents one bin obtained using the corresponding binning method. The color of a bin should be the same throughout if there is no binning error

Because MetaGen does not use the sequence information, the binning accuracy is not significantly affected when some of the species have highly similar sequences. However, MetaGen requires that the distribution of species in different samples be distinguishable. For example, as shown in Fig. 5, *Cupriavidus metallidurans* CH34 (green) and *Ralstonia eutropha* JMP134 (white), two species with highly similar sequences, are successfully separated by MetaGen but mistakenly binned together in MaxBin, CONCOCT, and MetaBAT.

#### Strain-level profiling

We studied the performance of MetaGen in distinguishing microbial strains using a mock data set with 57 *Escherichia coli* strains and 91 plasmids. The data set we generated using MetaSim contains 40 metagenomic samples, each with 2 million paired-end reads. MetaGen outperformed other reference-free binning methods we considered including CONCOCT, MetaBat, and MaxBin, as well as the reference-based method, CLARK, in strain-level discrimination. More specifically, the ARI for MetaGen was 0.50, which is significantly higher than that for CONCOCT (0.16). CLARK assigned all the contigs to one bin because the lowest taxonomy rank that CLARK can reach is at the species level. MetaBat and MaxBin also failed in strain-level profiling by binning all 57 *E. coli* strains into one bin (MetaBat) or two bins (MaxBin). The comparison results are summarized in Table 1.

**Table 1:** Adjusted rand index, precision, and recall of CLARK, MetaGen, MaxBin, CONCOCT, and MetaBAT evaluated on the simulated metagenomic community with 57 *Escherichia coli* strains

	MetaGen	MaxBin	CONCOCT	MetaBAT	CLARK
Adjusted rand index	0.50	0.01	0.16	0.00	0.00
Recall	0.65	0.86	0.80	1.00	1.00
Precision	0.81	0.16	0.48	0.13	0.12

More importantly, we found that MetaGen can also be applied to strain-level profiling, a promising and burgeoning area that is attracting a significant amount of attention. Unlike the reference-based strain-level profiling tools [23–25], which classify strains based on their sequence similarity to a strain-level reference genome, MetaGen can provide a fairly accurate strain-level profile without using reference genomes. Meanwhile, compared to the recent reference-free strain-profiling tools, ConStrains [26] and WG-FAST [27], which depend on single-nucleotide polymorphism to recover the strain profiles, MetaGen uses the sample profile to profile different strains and it requires a lower sequencing depth. In the data simulated under the same settings as [26], we found that MetaGen outperformed ConStrains in modified Jenson–Shannon divergence, a measure proposed in [26] to justify the profiling error. The modified Jenson–Shannon divergence was 0.04 for MetaGen and 0.26 for ConStrains. We did not compare MetaGen with ConStrains in distinguishing the 57 *E. coli* strains and 91 plasmids because ConStrains requires 10× coverage in at least one sample. This requirement was not satisfied by the 57 *E. coli* strains mock data set, which had only about 1.5× average coverage.

#### Binning results for a complex community

To investigate the effectiveness of MetaGen in analyzing complex metagenomic communities with a limited number of samples, we simulated ten metagenomic samples, each with 545 genomes and 439 plasmids based on the most abundant species identified by CLARK in the 269 gut metagenomic samples from [28, 29]. The relative abundance of each species in the ten samples was generated by the CLARK-estimated relative abundance from ten randomly selected samples in [28, 29] to mimic the real relative abundance. Summarized in Table 2 are the ARIs for MetaGen, CONCOCT, MaxBin, MetaBat, and CLARK. MetaGen achieved a higher binning accuracy compared to all the reference-free binning methods in comparison, but it had a lower accuracy compared to the reference-based method, CLARK.

**Table 2:** Adjusted rand index, precision, and recall of CLARK, MetaGen, MaxBin, CONCOCT, and MetaBAT evaluated on the complex metagenomic community with 545 genomes and 439 plasmids

	MetaGen	MaxBin	CONCOCT	MetaBAT	CLARK
Adjusted rand index	0.67	0.51	0.42	0.07	0.86
Recall	0.89	0.73	0.86	0.79	0.96
Precision	0.76	0.65	0.53	0.40	0.90

#### Reference-free estimation of relative abundances

**Fig. 6:**
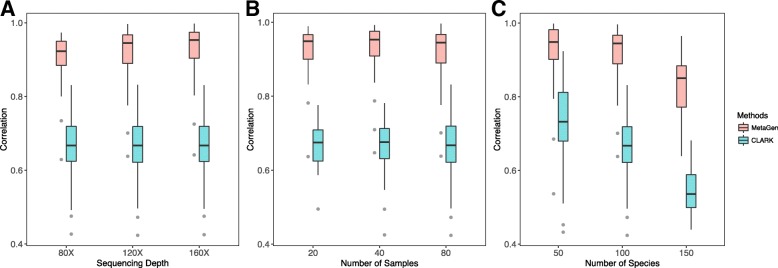
Box plot of the Pearson correlation coefficient between the estimated relative abundance (across different species within one sample) and the underlying truth. **a** The comparison is applied to the metagenomic data sets for different sequencing depths for 80 samples and 100 species. **b** The comparison is applied to the metagenomic data sets for different numbers of samples with 120× sequencing depth and 100 species. **c** The comparison is applied to the metagenomic data sets for different numbers of species with 120× sequencing depth and 80 samples

MetaGen provided an estimate of the relative abundance of the microbial species in each sample without utilizing any reference information. Compared to the reference-based methods, which estimate the relative abundance of each species using the proportion of reads from its genome showing up in each sample [30, 31], MetaGen estimates the relative abundance using the estimated sample profile for each bin (see Eq. 7 in ‘Methods’). To compare the relative abundance estimated by each tool, we used Pearson correlation coefficients [32] to characterize the overall relationship between the estimated relative abundance (across different species within one sample) and the underlying truth. We did the comparisons for all nine simulated data sets with varying sequencing depths, numbers of samples, and numbers of species. As shown in Fig. 6, the accuracy of the relative abundances estimated by MetaGen is significantly higher than those estimated by CLARK. Even for data with a very low sequencing depth (1× per sample), MetaGen demonstrated a high accuracy with an average correlation of 0.908 between the estimated relative abundance and the truth.

#### Some other factors relevant for estimation accuracy

Some minor but essential issues were also considered in our simulation. We first compared the binning accuracy of MetaGen to other candidate methods when some species were missing in certain samples. In this simulation, only 50 or 75 out of 100 species were randomly selected for each sample. The binning accuracy is plotted in Additional file 1: Figure S11, indicating that MetaGen was not affected by missing species.

We then tested how the binning accuracy is affected by using different genome assemblers, such as MegaHIT [33] and Ray [34]. Additional file 1: Figure S14 plots the ARI, recall, and precision of all five binning methods under consideration for Ray and MegaHIT, respectively. Clearly, CLARK, MetaGen, and MetaBAT performed marginally better using Ray while CONCOCT performs marginally better using MegaHIT. The binning accuracy of MaxBin was significantly better for MegaHIT compared to Ray. Compared to the other methods, MetaGen was least affected by the use of different assemblers.

### Metagenomic analysis of inflammatory bowel disease

Inflammatory bowel disease (IBD) is an idiopathic disease caused by humans’ dysregulated immune responses to their intestinal microbiota. IBD can cause abdominal cramps, bloody diarrhea, fever, and weight loss, and may also increase the risk of colon cancer. Each year, about 600 000 Americans suffer from one of the two IBD subtypes: ulcerative colitis (UC) and Crohn’s disease (CD). It was recently shown in [35] that IBD is closely related to aberrant interactions between gut microbial species and the host’s immune system.

Qin et al. [28] collected gut microbial DNA samples from 124 European individuals, including 25 IBD patients. The DNA samples were sequenced using Illumina Genome Analyzer with 576.7 Gb paired-end reads generated. Using MetaGen, we inferred that at least 2150 clusters/species (see Additional file 1: Figure S24) were presented in the samples, much more than the 155 species identified in [28] using a reference-based method. The significant difference between the two results is mainly caused by the limited availability of reference bacterial species. In fact, only 6.54*%* of the total contigs can find a closely matched reference genome in the National Center for Biotechnology Information (NCBI) nucleotide database. The scale of the number of species predicted by MetaGen is also consistent with the conjecture made in [28]. For the contigs that can be mapped to reference genomes, we found that MetaGen achieved a high binning accuracy with precision 0.937 and recall 0.753. We did not compare our method to other reference-free binning methods for this study because the data set was too large for other methods to obtain results using the computing resources we had access to.

**Fig. 7:**
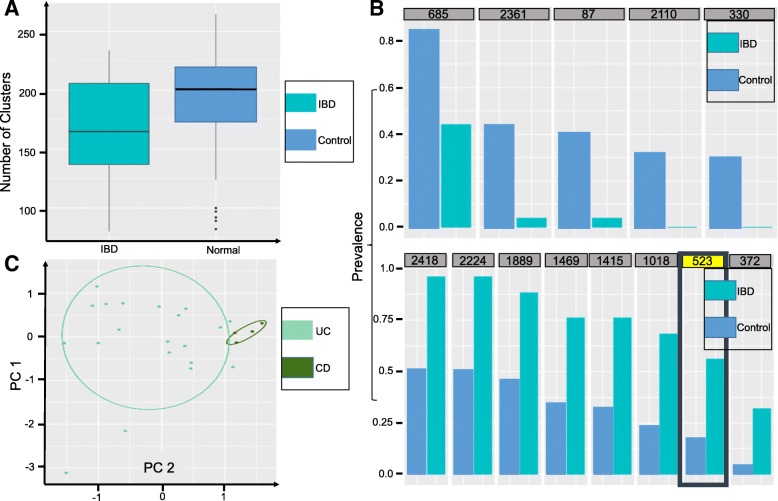
**a** Box plots of the number of significant species in each individual in the IBD and control groups, respectively. **b** Upper panel: Prevalence of the five highly enriched species in the individuals in the control group relative to the IBD patients. Lower panel: Prevalence of the eight highly enriched species in the IBD patients relative to the individuals in the control group. **c** The projection of the four CD patients and 21 UC patients along the first two principal component directions of the relative abundances of their microbial species. CD Crohn’s disease, IBD inflammatory bowel disease, PC principal component, UC ulcerative colitis

Figure 7 a shows box plots of the number of *significant microbial species* (see ‘Methods’ for a definition) found in each individual in the IBD and control groups, respectively, indicating that the biodiversity of microbiota in IBD patients is significantly lower than that in individuals in the control group (*p*-value = 0.03). This was also observed in [29] and [36]. By testing the 561 microbial species that were shared by at least ten individuals, we found that five species were significantly less common and eight species were significantly more common in IBD patients with the false discovery rate controlled at under 5% [37]. Among the eight species that are more commonly seen in IBD patients, we found that 13 of 25 contigs in one bin (highlighted by the black box in the lower panel of Fig. 7 b) could be mapped to a bacterial strain *Bacteroides fragilis* HMW 615 with more than 99% identity. Among the 13 contigs, six were mapped to *Bacteroides fragilis* HMW 615 with 100% identity.

Based on large-scale metagenomic data sets, predictive models using machine-learning tools have revealed good predictive capabilities for different phenotypes, such as disease state [38], plant productivity [39], and environmental factors [40]. To investigate whether the microbial composition estimated by MetaGen can be used for disease prediction, we built a logistic regression model with LASSO penalty [41] to classify the IBD and control subjects using the relative abundance (see Eq. 7 in ‘Methods’) of the clusters inferred by MetaGen as features. The cross-validation (CV) procedure was used to assess the classification accuracy. The overall prediction power of the logistic regression model is quite significant, with a tenfold CV misclassification rate of 0.089 (precision 0.938, recall 0.600, and area under the curve (AUC) 0.967). The number of misclassifications for the IBD group was one and for the control group it was ten. We further zoomed in to investigate the difference in gut microbiota between two types of patients, CD and UC, which are not readily separable using existing medical techniques [42]. Figure 7 c shows the projection of the 25 IBD subjects onto the space formed by their first and second principal components, which shows a clear separation between the two IBD subtypes.

### Metagenomic analysis of type 2 diabetes

Type 2 diabetes (T2D) is the most prevalent endocrine disease. It involves a long-term metabolic disorder influenced by both genetic and environmental factors [43]. Qin et al. [29] sequenced gut microbial DNA samples from 71 Chinese T2D patients and 74 Chinese individuals unaffected by T2D using Illumina Genome Analyzer and obtained 3.3 million genes based on 378.4 Gb paired-end reads. They could not obtain taxonomy assignments and the corresponding microbial distribution estimations using a reference-based binning method because only 8.89*%* of the contigs can be mapped to reference genomes. We re-analyzed this data set using MetaGen and identified 2450 species clusters (see Additional file 1: Figure S25).

Using Fisher’s exact test with the false discovery rate controlled at 5%, we found that two clusters were more abundant in the control group than in the T2D group (see Additional file 1: Figure S26). The majority of contigs in one of the clusters can be mapped to the butyrate-producing bacteria, *Roseburia intestinalis*, which has been shown in [44] to have an immuno-metabolic effect and is, thus, significantly less abundant in T2D patients. This finding also validates the conjecture made in [29] that beneficial bacteria are universally lost in the T2D gut. We also tested to differentiate T2D patients from the control group by building a classifier using the subjects’ microbial distributions and the LASSO-logistic regression method used in the previous section. We observed that the tenfold CV classification error rate was 0.317 (precision 0.687, recall 0.648, and AUC 0.754). We further validated the classification accuracy using an independent data set from [29] with 98 T2D patients and 99 controls, and obtained a misclassification error of 0.350 (precision 0.653, recall 0.646, and AUC 0.699), which is highly significant. Although the prediction accuracy is not yet ideal, our study of the T2D metagenomic data showed that an individual’s microbial composition estimated in a reference-free way can be significantly predictive of the individual’s disease status.

### Metagenomic analysis of obesity

Obesity is a growing epidemic worldwide and has a significant negative impact on human health. Obese people have significantly higher risks for various diseases, such as high blood pressure, stroke, heart disease, diabetes, cancer, gallstones, etc. Despite its clinical importance, the causes of obesity and possible therapeutic options for curing it remain poorly understood. Recent studies have found that some bacteria in the human gut can disrupt the metabolic/energy homeostasis [2, 45], and the bacteria’s interactions with the host’s genes [46] are closely associated with the host’s obesity level. It is, thus, expected that understanding the bacterial compositions of metagenomic samples from human guts may be key to understanding obesity.

In [1], DNA samples were extracted from the feces of 18 human subjects belonging to six families, each of which includes a pair of twins and their maternal parent. After pre-processing (see Additional file 1: SI Note), we obtained 25 383 contigs. For each contig, we searched the NCBI nucleotide database and used TAXAassign (https://github.com/umerijaz/TAXAassign) to assign it to a taxonomic group. Only 29% of the contigs could be assigned at the species level and 54% could be assigned at the phylum level. Roughly 46% of contigs could not be mapped to any reference genomes even at the phylum level. Thus, reference-free binning methods are highly desirable for this data.

**Fig. 8:**
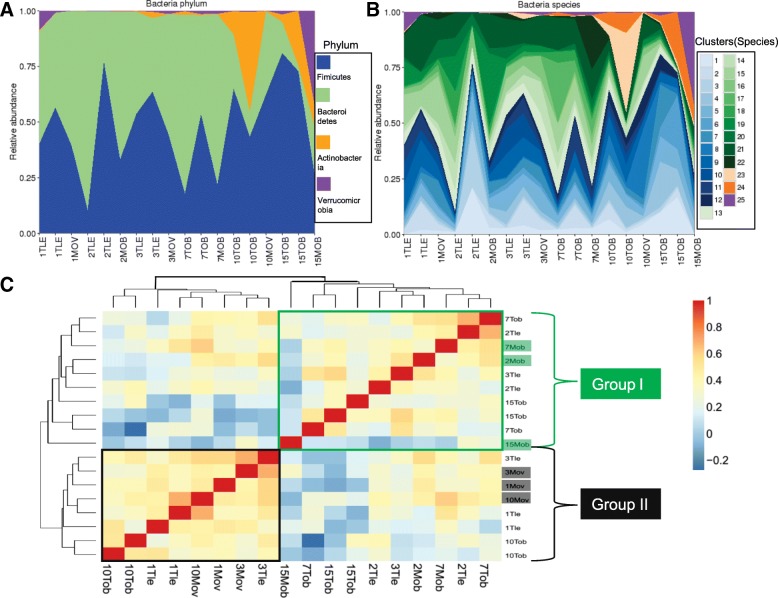
**a** Relative abundances of Firmicutes, Bacteroidetes, Actinobacteria, and Verrucomicrobia phyla estimated by MetaGen. **b** Relative abundances of the 25 species mapped to one of the four aforementioned phyla. Clusters 1 to 12 are species in Firmicutes, clusters 13 to 22 are species in Bacteroidetes, clusters 23 to 24 are species in Actinobacteria, and cluster 25 is a species in Verrucomicrobia. **c** Heat map of the correlation of the relative abundance for the 18 individuals (samples). The samples are clustered by hierarchical clustering using complete linkage functions. In all the plots, a subject’s ID can be parsed into three parts: the family ID (1–6), twin or mother (T, M), and body mass index (lean, overweight, or obese)

Using MetaGen, we identified 56 bins/species (Additional file 1: Figure S27) and estimated their relative abundances across samples. For the contigs that have species-level reference genomes, we compared MetaGen with CONCOCT using the reference-based binning results as a gold standard. We observed that the results of MetaGen were closer to the reference-based binning results (with ARI of 0.746) than those of CONCOCT (with ARI of 0.592). In Fig. 8 a, we compared the estimated relative abundances to those published in [2] at the phylum level. MetaGen can accurately estimate the relative abundances of the four most enriched phyla: Firmicutes, Bacteroidetes, Actinobacteria, and Verrucomicrobia. Figure 8b provides a more detailed relative abundance estimate at species level, an estimate that could not be obtained in [2] due to the limitations of the reference-based binning methods.

Figure 8c shows all pairwise Pearson correlations of the relative abundance for the 18 individuals. Using hierarchical clustering, we obtained two major clusters. Group I includes three families, in which all the mothers were obese although the children were either obese or lean. In contrast, all mothers in group II were overweight. Members of one family only were split into the two clusters. The correlation analysis suggests that the microbial distribution of the mother is associated with her body mass index (BMI) status and also plays a key role in shaping up the microbial distribution of her children. To test the predictive power of the microbial distribution of the identified species for an individual’s BMI status, we fitted a LASSO-logistic regression model using the relative abundances as predictors and the individual’s BMI status as the response. The leave-one-out CV error rate of the resulting model was 0.33.

## Discussion

We proposed a new method, MetaGen, for estimating species compositions in multiple metagenomic samples without any prior knowledge of either reference microbial genomes or the actual microbial distributions of the samples. MetaGen is, thus, a completely reference-free metagenomic procedure and is especially useful for analyzing new and foreign microbial samples. As demonstrated by our simulation studies, MetaGen can handle data with fairly low sequencing coverage, which can be extremely challenging with the currently available methods for metagenomic analysis. When a reference genome is available for some of the microbial species, we recommend the use of MetaGen together with reference-based methods as a safeguard against possible false positives.

As a trade-off for having no reference genomes, MetaGen requires multiple samples (preferably ≥10) and imposes a key *differential abundance* assumption, i.e., the abundance patterns of microbial species across multiple samples should vary appreciably. This assumption is clearly confounded with sequencing depth in the study: by increasing the sequencing depth, one can recognize more species, as is true for all other available methods. The differential abundance assumption can be satisfied in most metagenomic studies related to human health, such as the study of microbial distributions in the human gut and the study of human pathogens in a biothreat attack. When the number of bacterial species is extremely large, many low-abundance species will have low coverage and cannot be detected. This limitation can be overcome by performing a screening step to trim the contigs with very low coverage.

## Conclusions

Accumulating evidence suggests that the microbial ecosystems play a crucial role in human health. However, compared to the huge amounts of medical research on human cells, our understanding of the microbial ecosystems is very limited: the biodiversity of them is not completely understood, not to mention their interactions with the human host. In this paper, we proposed a reference-free metagenomic binning method, MetaGen, which not only identifies bacteria species but also quantifies their distributions. With the growing number of the metagenomic samples being sequenced, we believe that our effort can benefit both the computational biologist and the experimental biologist in studying the changes of the microbial ecosystems, detecting pathogens and reducing the diagnostic error in microbial-related human diseases.

The MetaGen pipeline is open-source software, and is freely available at the URL https://github.com/BioAlgs/MetaGen.

## Methods

### Connection with non-negative matrix factorization

The sample-profile-based binning problem can also be solved by a non-negative matrix factorization (NMF) algorithm, of which the EM algorithm can be viewed as a principled generalization. If the information is strong enough so that random errors and fluctuations can be ignored, the (*i*,*j*)th entry of RCMM, *x*_*ij*_, is just the *theoretical* number of reads that are mapped to contig *i* in sample *j*, which should be equal to the number of short reads that one copy of contig *i* can produce multiplied by the number of copies of contig *i* in the *j*th sample.

If we assume that the contig is long enough so that it belongs only to one species, we can rewrite the RCMM **X** as the product of a signature matrix *M* and the total abundance matrix *E*, where the (*i*,*k*)th entry of *M* is the number of reads that a single copy of contig *i* in species *k* can produce (it is zero if the *k*th species does not contain contig *i*), and the (*k*,*j*)th entry of *E* represents the number of species *k* in sample *j*. Thus, we can obtain an estimate of both *M* and *E* simultaneously by minimizing ||*X*−*M* *E*||_*F*_, where ||·||_*F*_ denotes the Frobenius matrix norm. Note that if we normalize each row of *E* to sum to one, we get the sample profile matrix *A*, i.e., *E*=*D* *A*, where *D* is a diagonal matrix with *d*_*ii*_ indicating the total number of counts for contig *i* in the pooled sample. Based on extensive simulations, we observed that the NMF algorithm and the EM algorithm lead to very similar results empirically for given *K*. However, this NMF approach cannot account for the estimation uncertainty and also does not provide a principled way to determine the number of species *K*.

### Normalization to compare microbial distributions across samples

To compare microbial distributions across samples, we need to normalize the sample profiles of different species to control the between-sample library size (sequencing depth) variation and the genome length variation. Motivated by the definition of RPKM, which has been commonly used to normalize RNAseq data across samples and across genes, we first rescale the number of mapped reads for species *k* in sample *j*, i.e., $\hat {a}_{kj}{\sum \nolimits }_{\{i:\hat {z}_{i}=k\}}n_{i}$, where *n*_*i*_ is the total number of mapped reads on contig *i*, by a factor reflecting sample *j*’s library size, i.e., the total number of reads *T*_*j*_ in sample *j*, and by another factor estimating the genome length of each species, i.e., the sum of the lengths of all contigs for species *k*, say *L*_*k*_. To set the number in a comfortable range, we multiply the rescaled number by a constant 10^9^ and denote it by $\hat {b}_{kj}$:

7 $$ \begin{aligned} \hat{b}_{kj} =10^{9}\times \frac{\hat{a}_{kj}{\sum\nolimits}_{\{i:\hat{z}_{i}=k\}}n_{i}} {L_{k}T_{j}}, \end{aligned}  $$

where $\hat {a}_{kj}$ and $\hat {z}_{i}$ are obtained using our algorithm. We refer to $\hat {b}_{kj}$ as the *relative abundance* of species *k* in sample *j*. To compare the relative abundance in each sample, we recommend adding an additional step to correct the GC bias by using GCcorrect (R package) [47]. When a species has relative abundance $\hat {b}_{kj}\ge 0.1\%{\sum \nolimits }_{k=1}^{K}\hat {b}_{kj}$, we define the species to be a *significant microbial species* for sample *j*. Here, we use 0.1*%* as a convenient cutoff because the relative abundances that are lower than 0.1*%* may suffer from a much higher estimation error and, thus, be unreliable.

### Evaluating the binning results

To evaluate the estimated bins with true taxonomic groups, we define two groupings, **x**=(*x*_1_,…,*x*_*r*_) and **y**=(*y*_1_,…,*y*_*s*_), where *r* and *s* are the number of clusters for groupings **x** and **y**, respectively. Then we denote *n*_*ij*_ as the number of members that belong to both the *x*_*i*_ and *y*_*j*_ clusters (overlap). ARI is defined as

8 $$ \text{ARI} = \frac{{\sum\nolimits}_{i,j}\dbinom{n_{ij}}{2}-E} {\frac{1}{2}\left[{\sum\nolimits}_{i}\dbinom{r_{i}}{2}+{\sum\nolimits}_{j}\dbinom{c_{j}}{2}\right] - E},  $$

where $r_{i} = \sum _{j=1}^{s}n_{ij}$ is the number of members in *x*_*i*_ cluster, $c_j = \sum _{i=1}^{r} n_{ij}$ is the number of members in *y*_*j*_ cluster, and

$$E= \frac{\left[{\sum\nolimits}_{i} \dbinom{r_{i}}{2}{\sum\nolimits}_{j}\dbinom{c_{j}}{2}\right]}{\dbinom{N}{2}} $$

is the expected index.

*Precision* is defined as the clustering accuracy under the most favorable species label assignment for each cluster. That is, assuming that grouping **y** is the true species label, the precision can be expressed as

9 $$ \text{Precision} = \frac{{\sum\nolimits}_{i=1}^{r}\max(n_{i1},\dots,n_{is})}{N}.  $$

On the other hand, *recall* is defined as how well the best cluster for each species regroups all the cluster’s contigs. That is, assuming that grouping **y** is the true species label, recall is

10 $$ \text{Recall} = \frac{{\sum\nolimits}_{j=1}^{s}\max(n_{1j},\dots,n_{rj})}{N}.  $$

## Additional file

Additional file 1 Supporting Information. Contains all supplementary figures and supplementary tables. (PDF 1782 kb)
