# Supplementary material for: MetaGen: reference-free learning with multiple metagenomic samples
Source: Genome Biol. 2017 Oct 3;18:187. doi: 10.1186/s13059-017-1323-y (PMC5627425; doi:10.1186/s13059-017-1323-y)
Supplement: Additional file 1 — Supporting Information. Contains all supplementary figures and supplementary tables. (PDF 1782 kb) [file 13059_2017_1323_MOESM1_ESM.pdf]

Additional File 1
Supporting Information
Reference-free Learning with Multiple Metagenomic Samples

1 Simulated Data Sets

In our simulation studies, we generated reads using MetaSim [1] with Illumina 80-bp paired-end reads error model. To model the high variation of the bacteria distribution across different samples, we simulated the relative abundances of species in each sample from a Dirichlet distribution. The 150 microbial species used in all simulation settings are listed in Table **S1**. MetaGen, MaxBin and CLARK are based on contigs longer than 1000bp. MetaBAT is based on contigs longer than 1500bp, which is the algorithm's minimum length cut-off. In simulation settings 1-3, we considered the sequencing depth, the number of samples, and the number of species as three variables. In each setting, we fixed two variables and change the other one.

Simulation Settings 1-3:
In the first setting, we fixed the number of samples at 80 and the number of specie at 100, and changed the pooled sequencing depth as 80**x**, 120**x** and 160**x**, which is equivalent to 1**x**, 1.5**x** and 2**x** per sample. In the second setting, we fixed the pooled sequencing depth at 120**x** and the number of species at 100, and changed the number of sample as "20", "40" and "80". In the third setting, we fixed the pooled sequencing depth at 120**x** and the number of samples at 80, and changed the number of species as "50", "100" and "150". All the detailed binning results of MetaGen, CLARK, MaxBin, MetaBAT, and CONCOCT are shown in Figures **5** and **S2-S8**. The computing times are shown in Figure **S1**. The correlations between the estimated relative abundances and the true relative abundances is shown in Figure **6**.

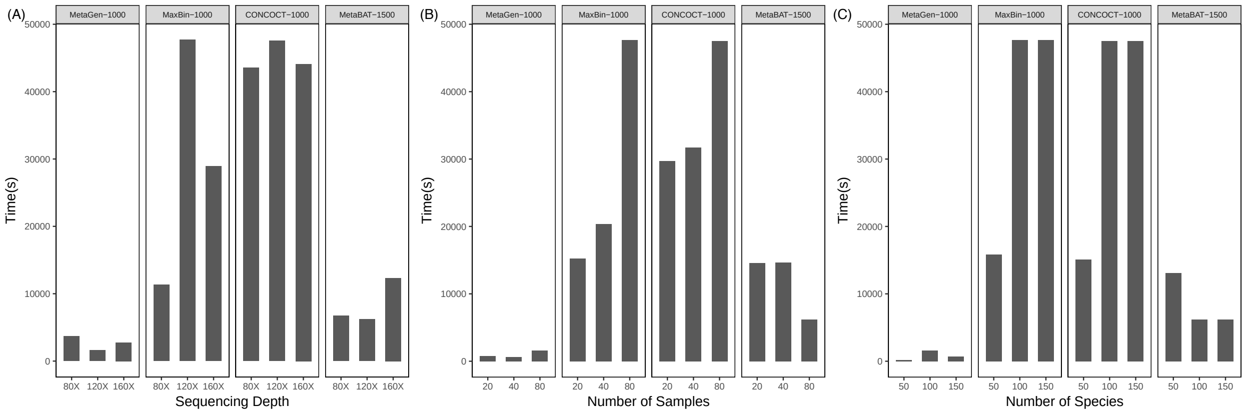

Figure S1: Computing times for MetaGen, MaxBin, CONCOCT and MetaBAT under different sequencing depths, different numbers of samples and different numbers of species.

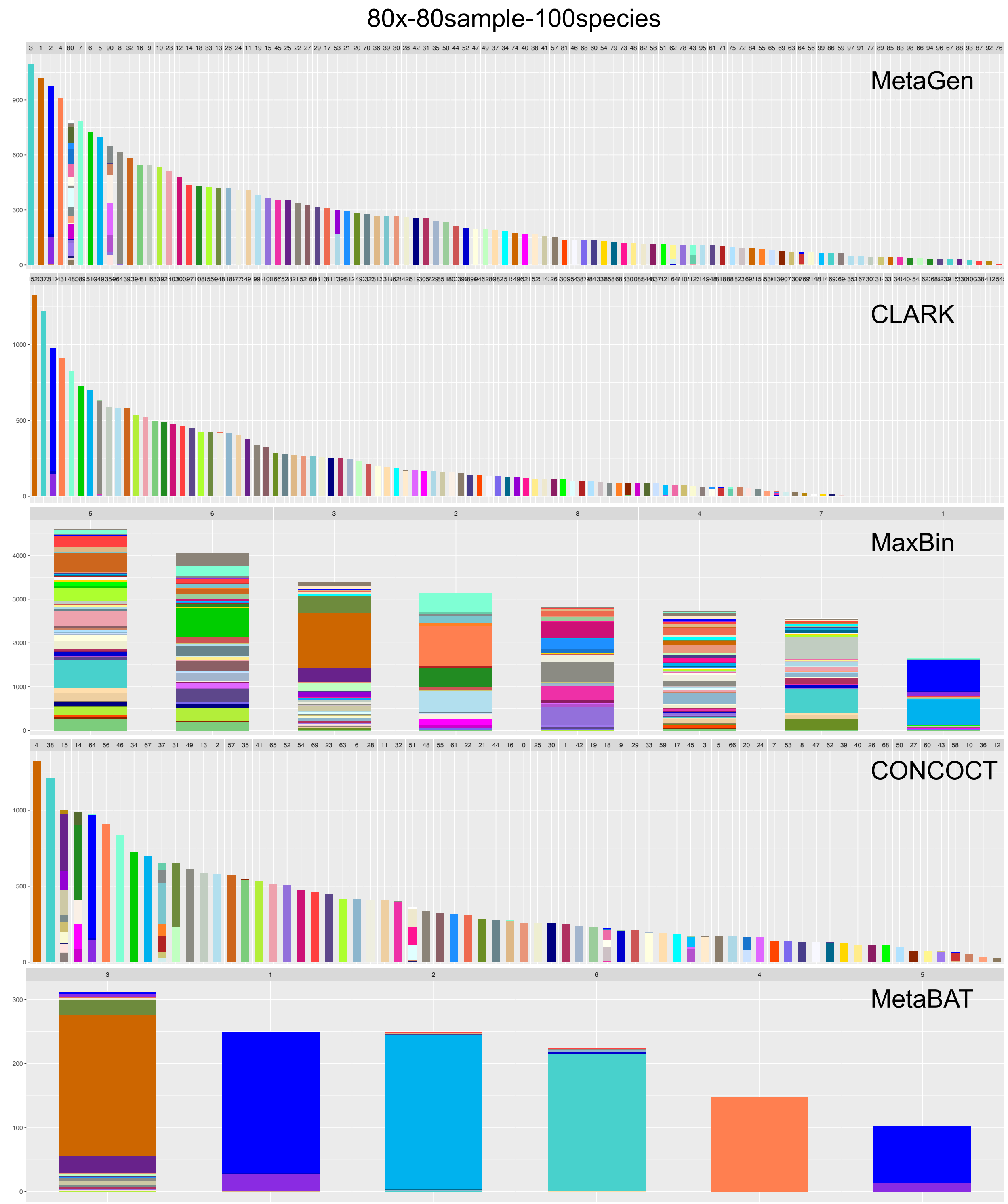

Figure S2: Binning results for MetaGen, CLARK, MaxBin, CONCOCT and MetaBAT with the pooled sequencing depth fixed at 80**x**, the number of samples at 80 and the number of species at 100. Color legends are given in **Figure S9**.

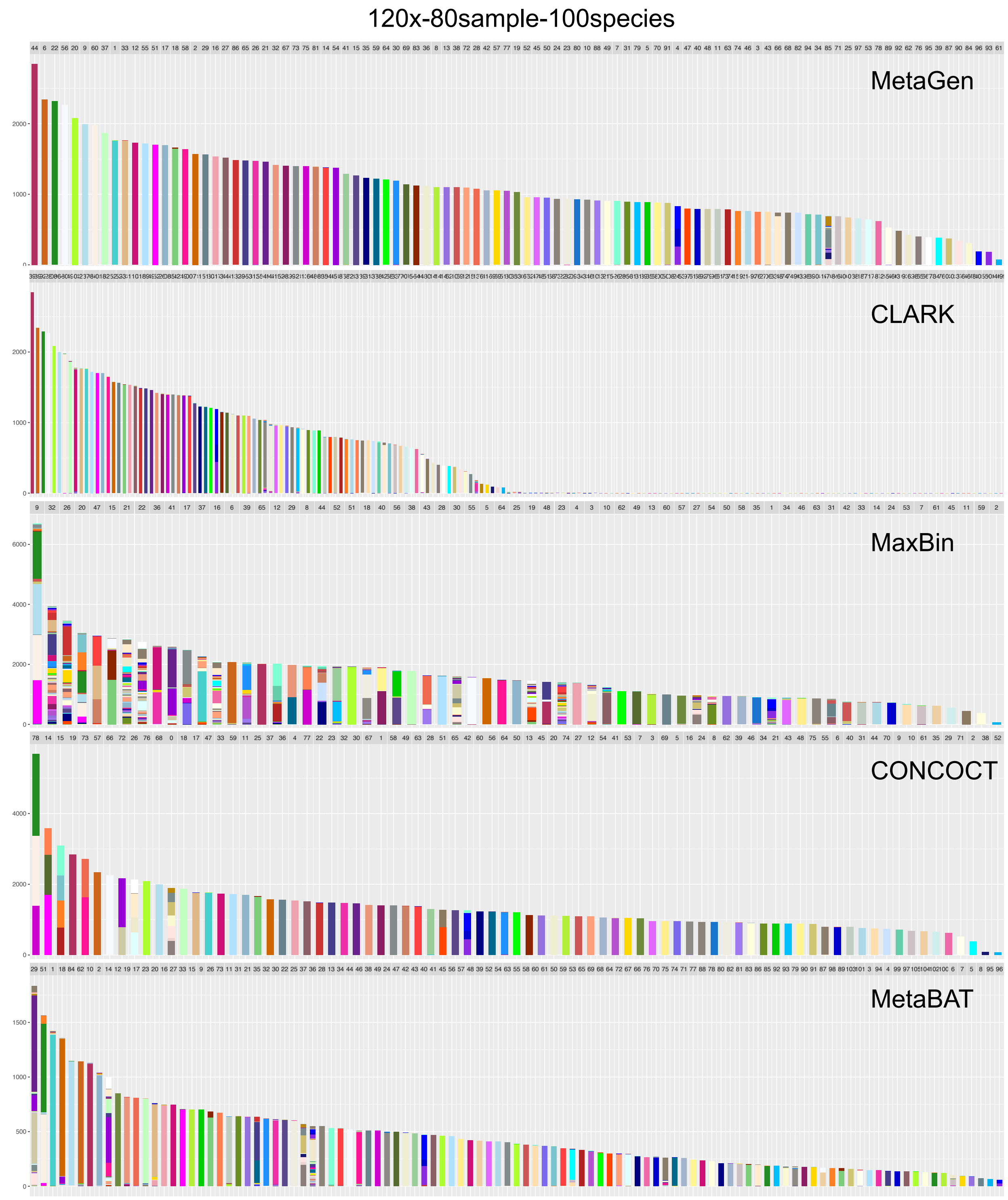

Figure S3: Binning results for MetaGen, CLARK, MaxBin, CONCOCT and MetaBAT with the pooled sequencing depth at 120**x**, the number of samples at 80, and the number of species at 100. Color legends are given in **Figure S9**.

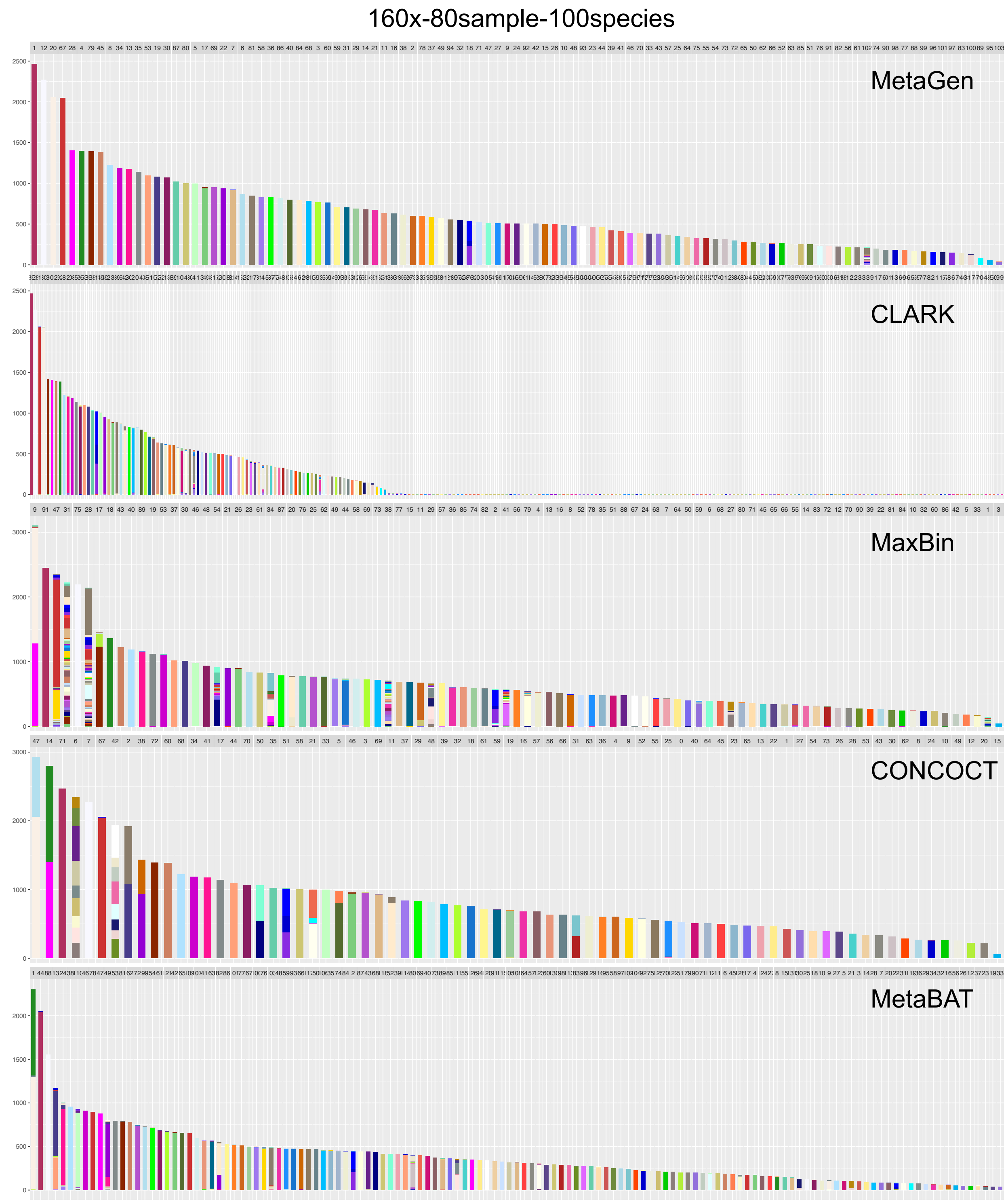

Figure S4: Binning results for MetaGen, CLARK, MaxBin, CONCOCT, and MetaBAT with the pooled sequencing depth at 160**x**, the number of samples at 80, and the number of species at 100. Color legends are given in **Figure S9**.

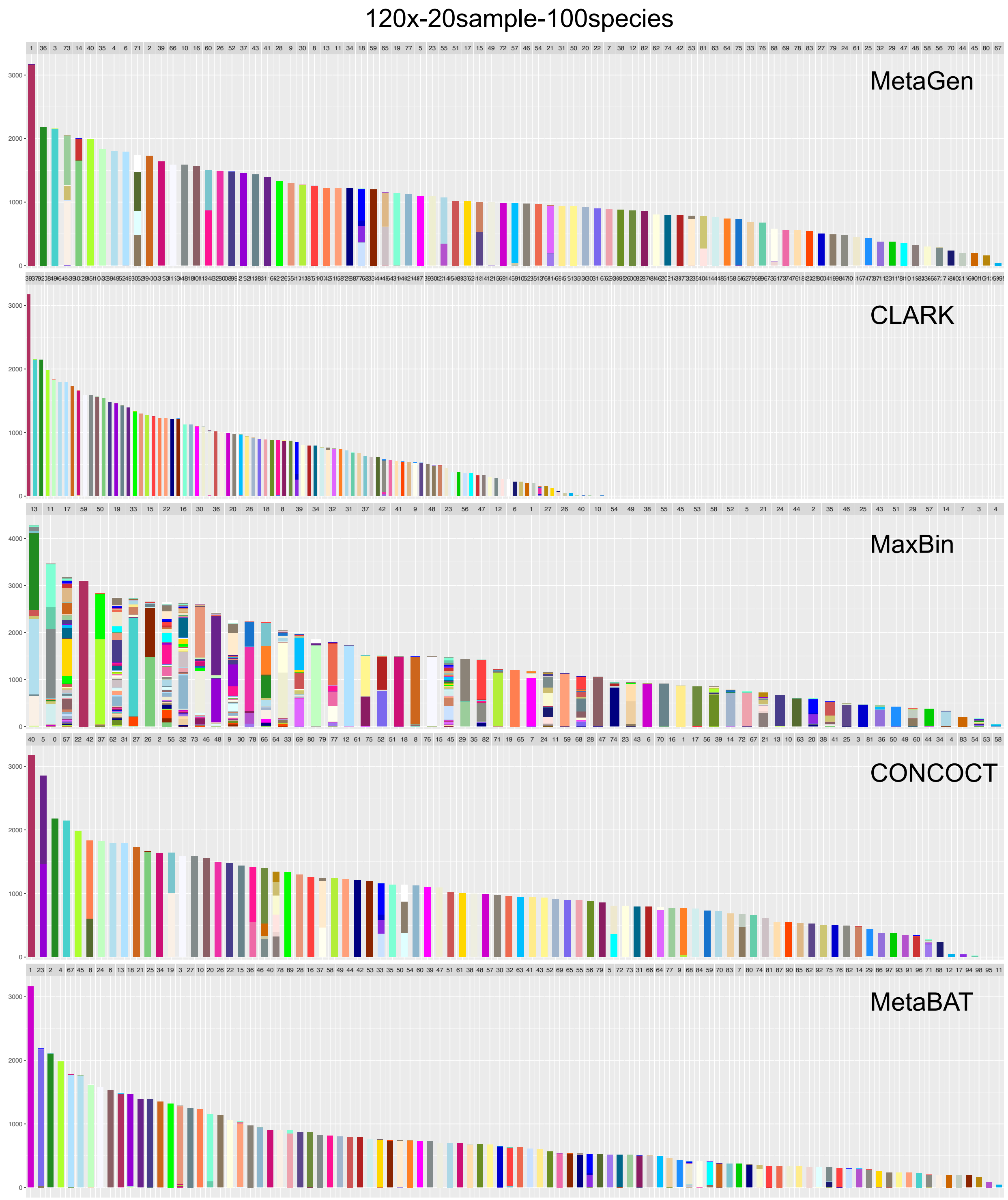

Figure S5: Binning results for MetaGen, CLARK, MaxBin, CONCOCT, and MetaBAT with the pooled sequencing depth at 120**x**, the number of samples at 20 and the number of species at 100. Color legends are given in **Figure S9**.

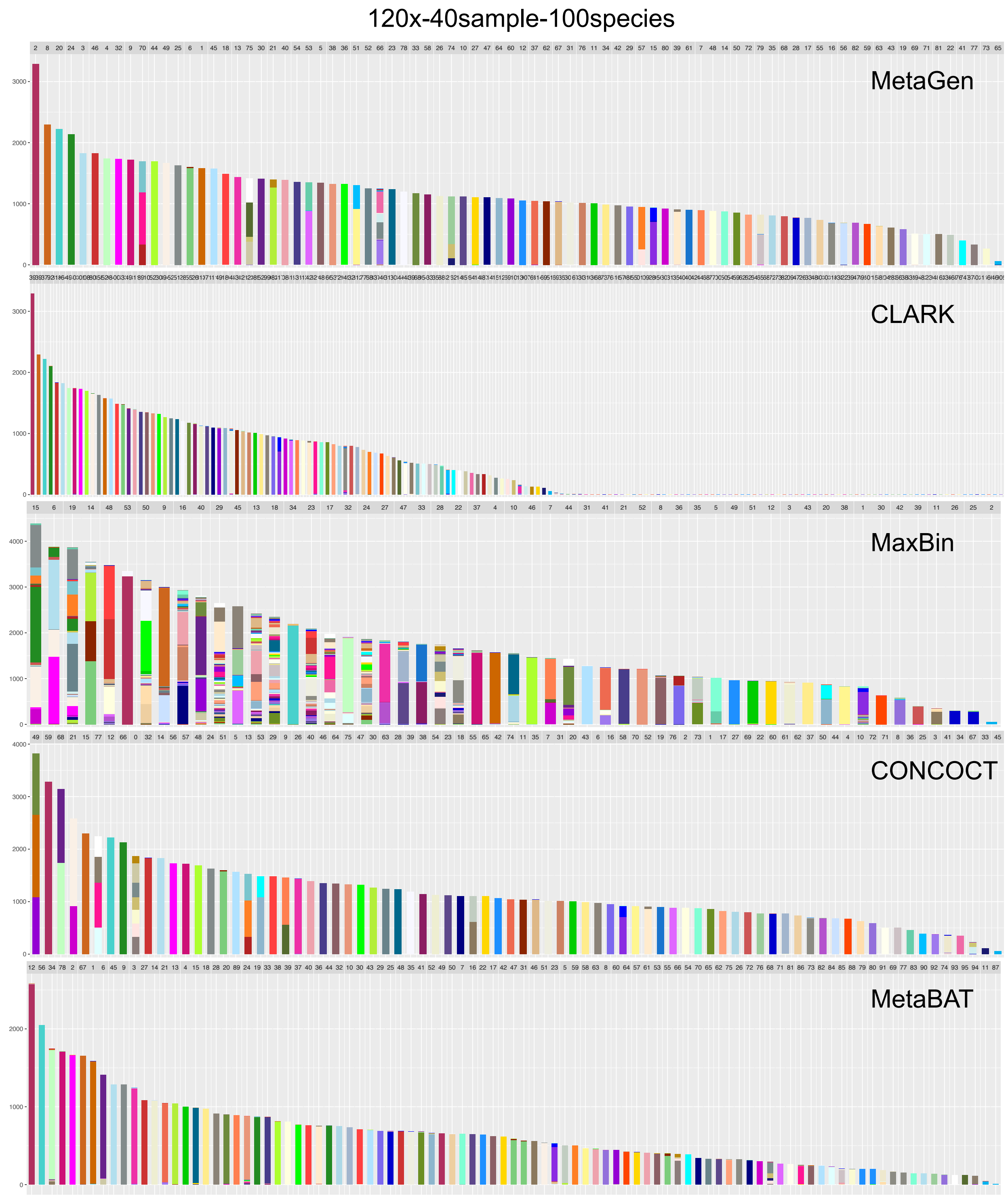

Figure S6: Binning results for MetaGen, CLARK, MaxBin, CONCOCT, and MetaBAT with the pooled sequencing depth at 120**x**, the number of samples at 40, and the number of species at 100. Color legends are given in **Figure S9**.

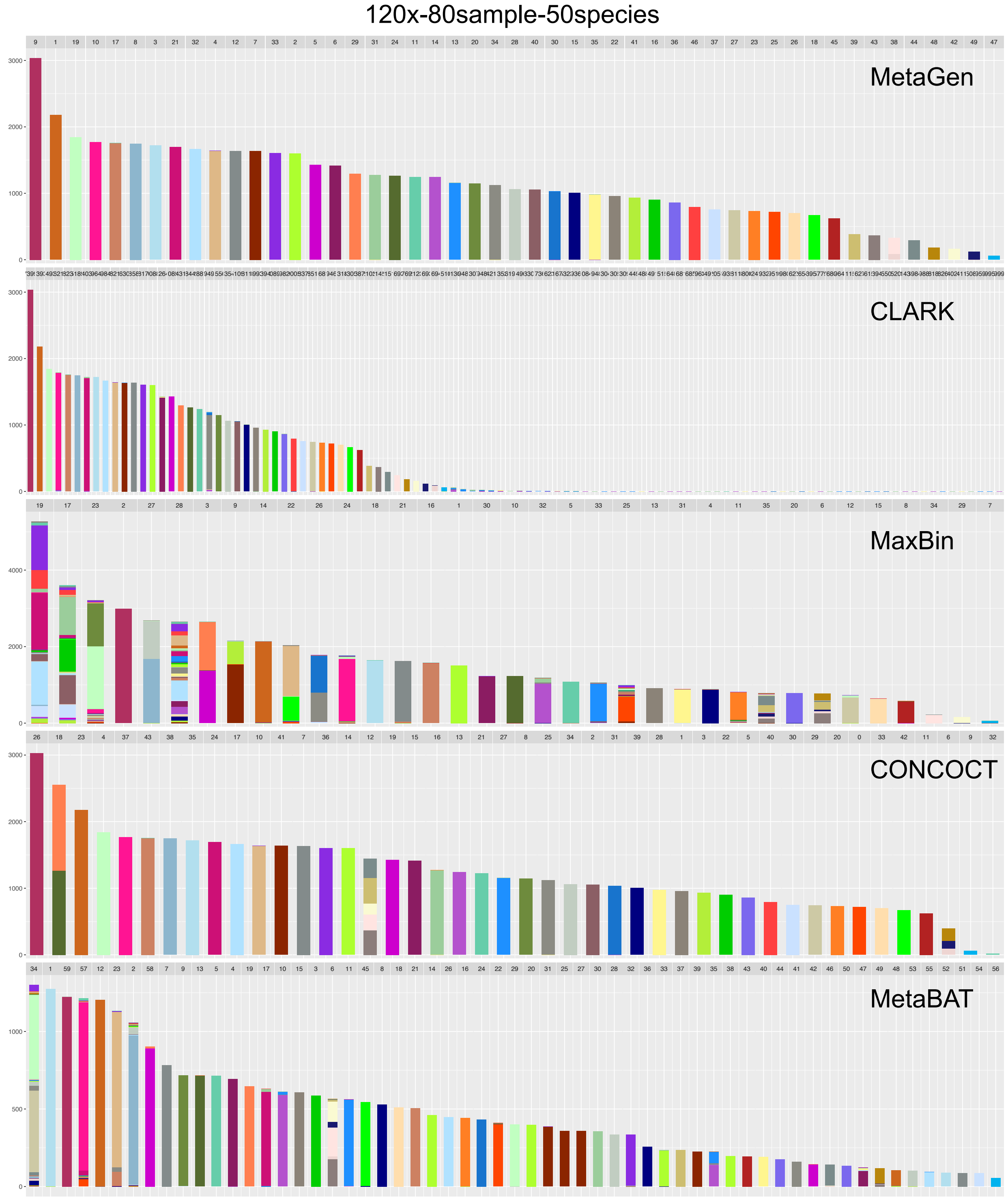

Figure S7: Binning results for MetaGen, CLARK, MaxBin, CONCOCT, and MetaBAT with the pooled sequencing depth at 120**x**, the number of samples at 80, and the number of species at 50. Color legends are given in **Figure S9**.

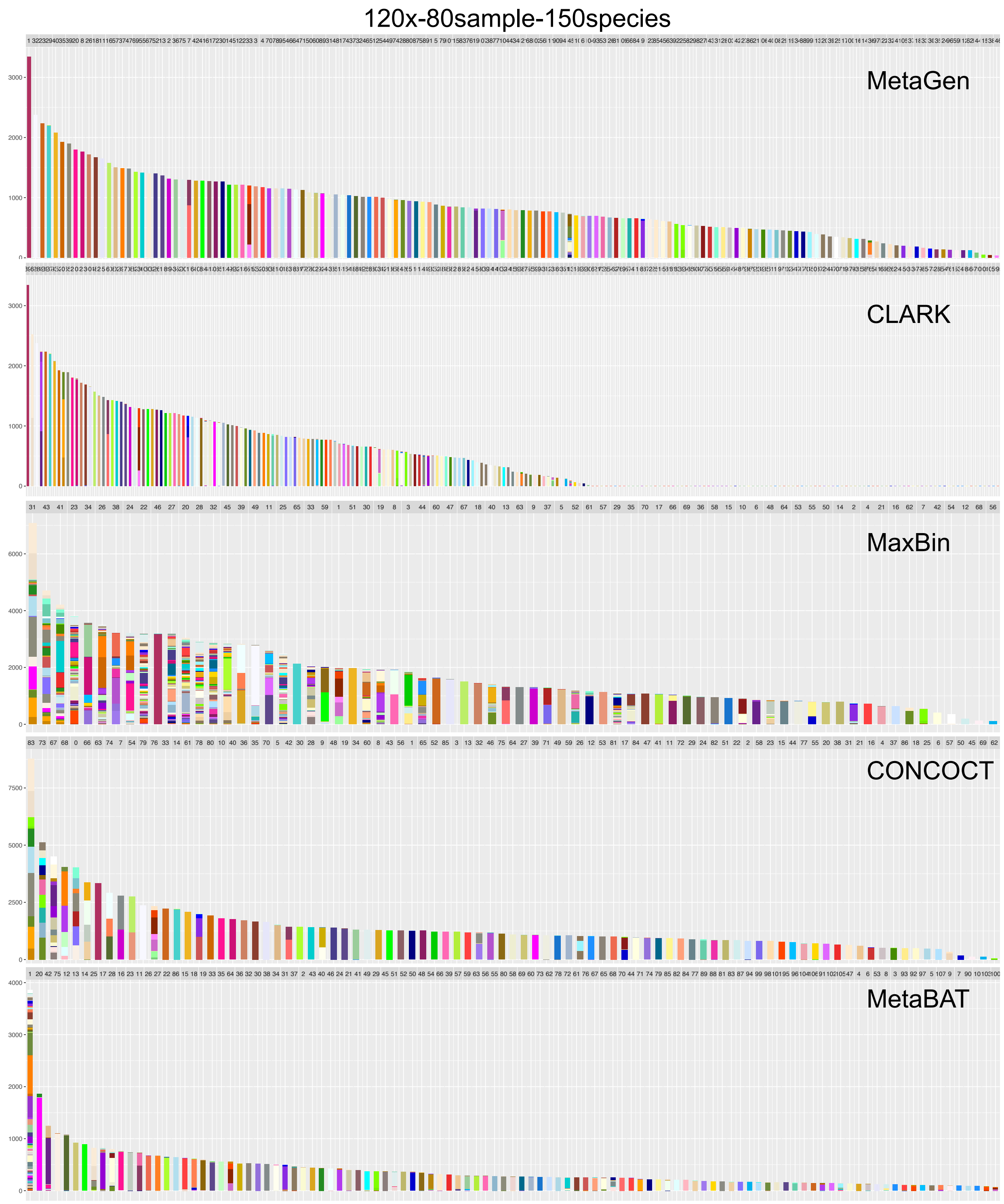

Figure S8: Binning results for MetaGen, CLARK, MaxBin, CONCOCT, and MetaBAT with the pooled sequencing depth at 120**x**, the number of samples at 80, and the number of species at 150. Color legends are given in **Figure S9**.

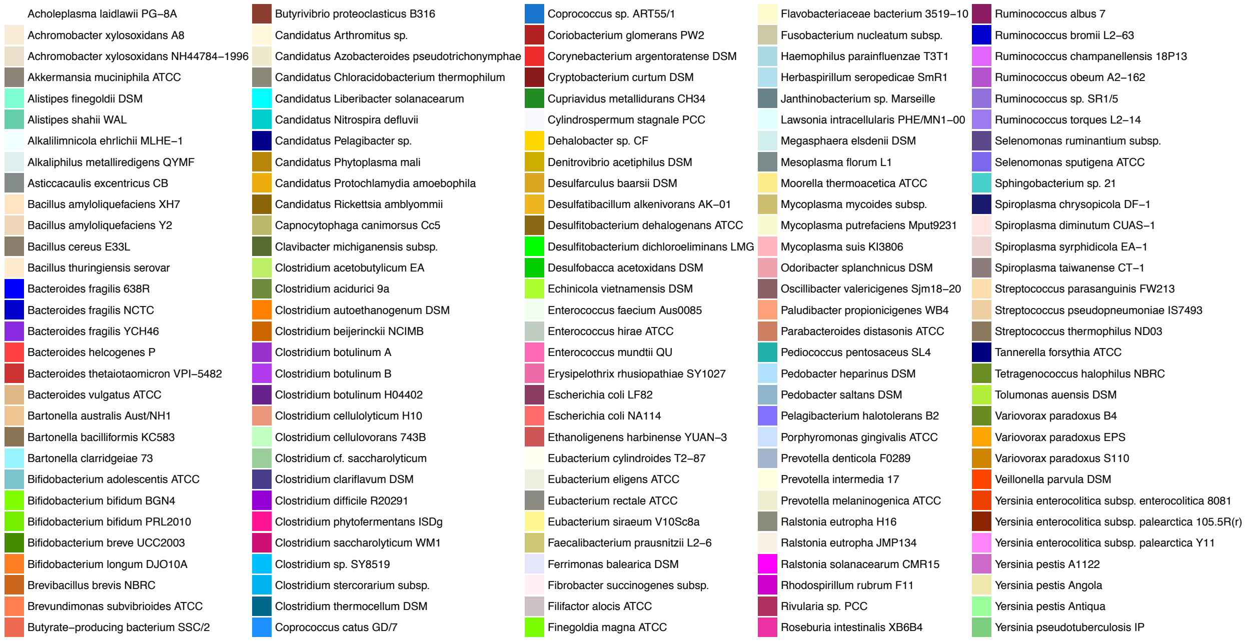

Figure S9: Color codes for all the 150 species used in Figures S1-S8.

Simulation Setting 4:

In this simulation setting, we first simulated a metagenomic community including 54 strains of *E. coli* (listed in Table **S2**). We generated 40 samples each with 2 million pair-end reads using MetaSim [1] with Illumina 80-bp paired-end reads error model.

Second, we simulated a 100**x** coverage of 100-bp paired-end Illumina reads of 6 ecoli strains (see Table **S2**) by ART simulator [2] with default settings for Illumina and library settings as -m 350 -s 50. These samples were further grouped together to simulate 6 samples using the relative abundance provided in (supplementary Table 1 in [3].)

Simulation Setting 5:

In this simulation study, we generated reads using MetaSim [1] with Illumina 80-bp paired-end reads error model. we generated 10 samples each with 25 million reads. To model the high variation of the bacteria distribution across different samples, the relative abundance of species in each sample was based on the 10 randomly selected real samples in our real examples. The histogram of the log-transformed relative abundance is shown in Figure **S10**. The 545 microbial species used in this simulation setting are listed in Table **S3**.

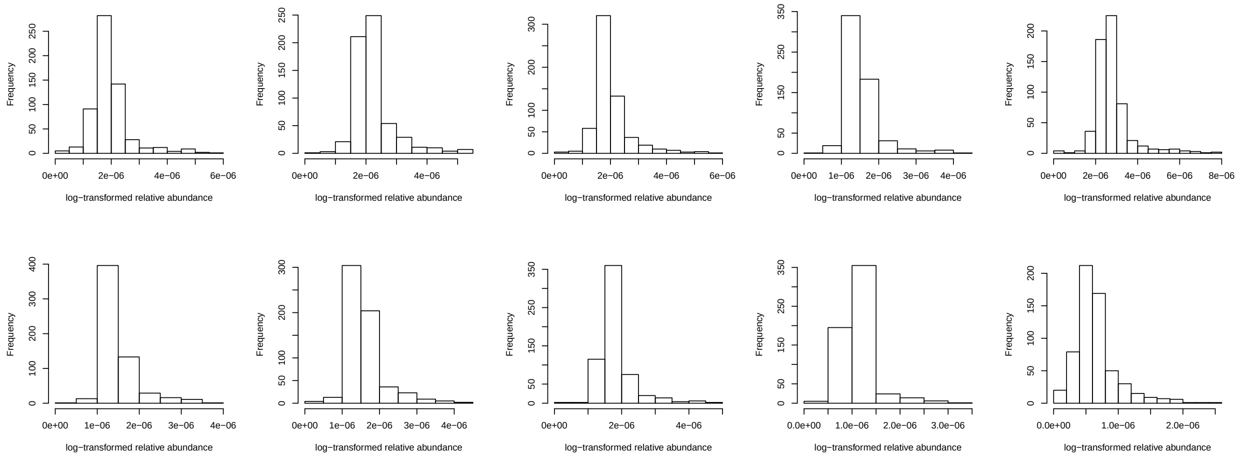

Figure S10: Histogram of the log-transformed relative abundance of different species for the 10 samples for the complex metagenomic data sets with 545 genomes and corresponding 439 circular elements.

Simulation Setting 6:

We fixed the pooled sequencing depth at 120**x**, the number of samples at 80, and the number of specie at 100, and changed the proportion of species in each sample from 50% to 100%. Performances of MetaGen, CLARK, MaxBin, MetaBAT and CONCOCT are shown in Figure **S11**. The detailed binning results of MetaGen, CLARK, MaxBin, MetaBAT, and CONCOCT are shown in

Figures **S12, S13, 5**.

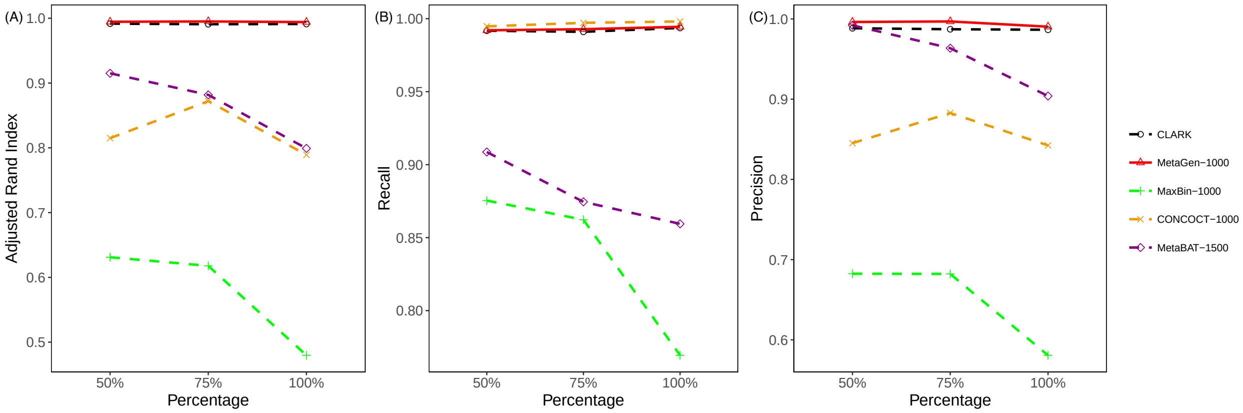

Figure S11: Adjusted Rand Index (A), Precision (B), and Recall (C) of CLARK, MetaGen, MaxBin, CONCOCT, and MetaBAT are evaluated on the simulated data sets with 50%, 75%, and 100% species, respectively, in each sample. All the three simulated data sets have 80 samples, 100 species, and the sequencing depth of 120**x**.

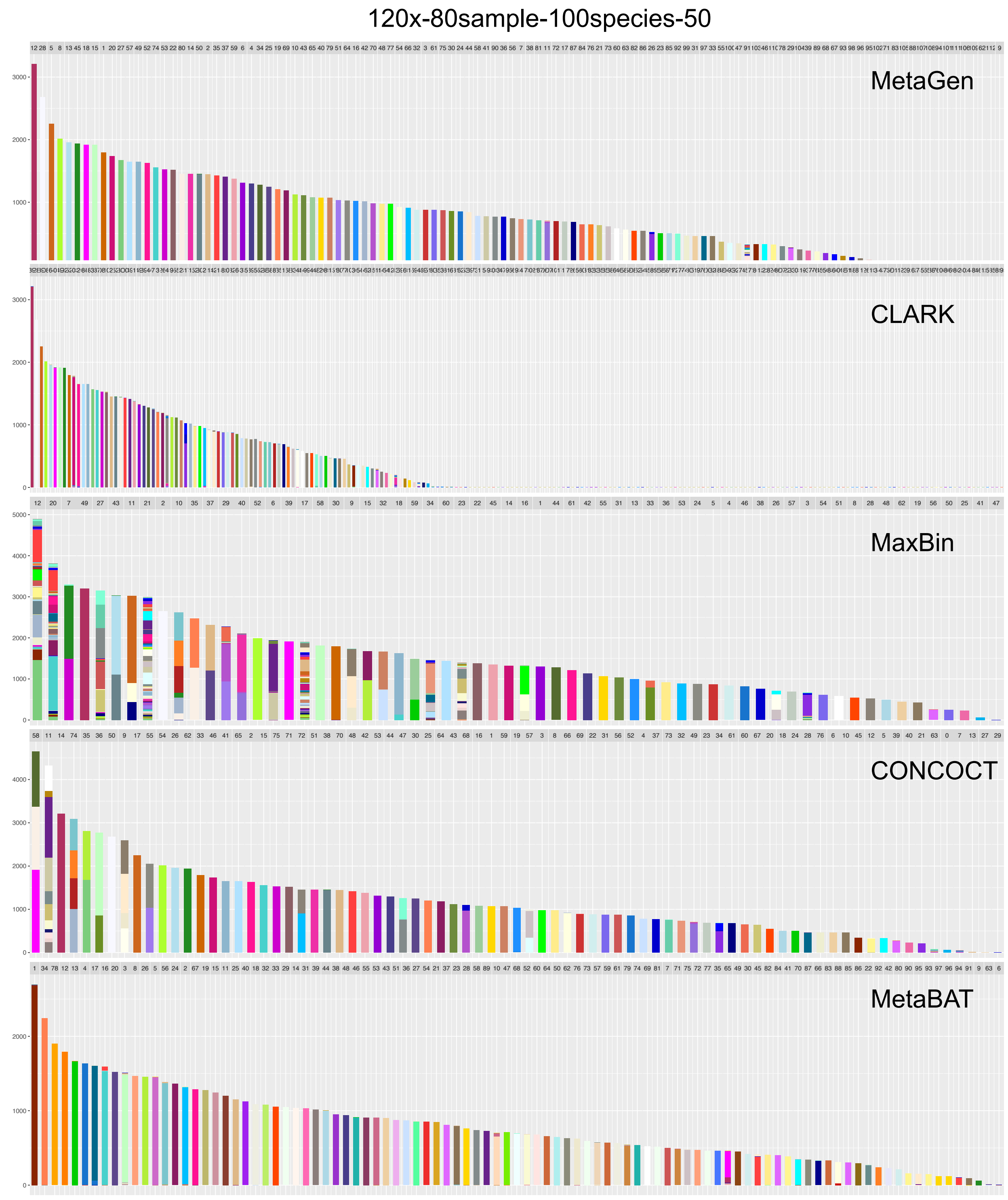

Figure S12: Binning results for MetaGen, CLARK, MaxBin, CONCOCT, and MetaBAT with the pooled sequencing depth at 120**x**, the number of samples at 80, and the number of species at 150, and with 50% of species in each sample. Color legends are given in **Figure S16**.

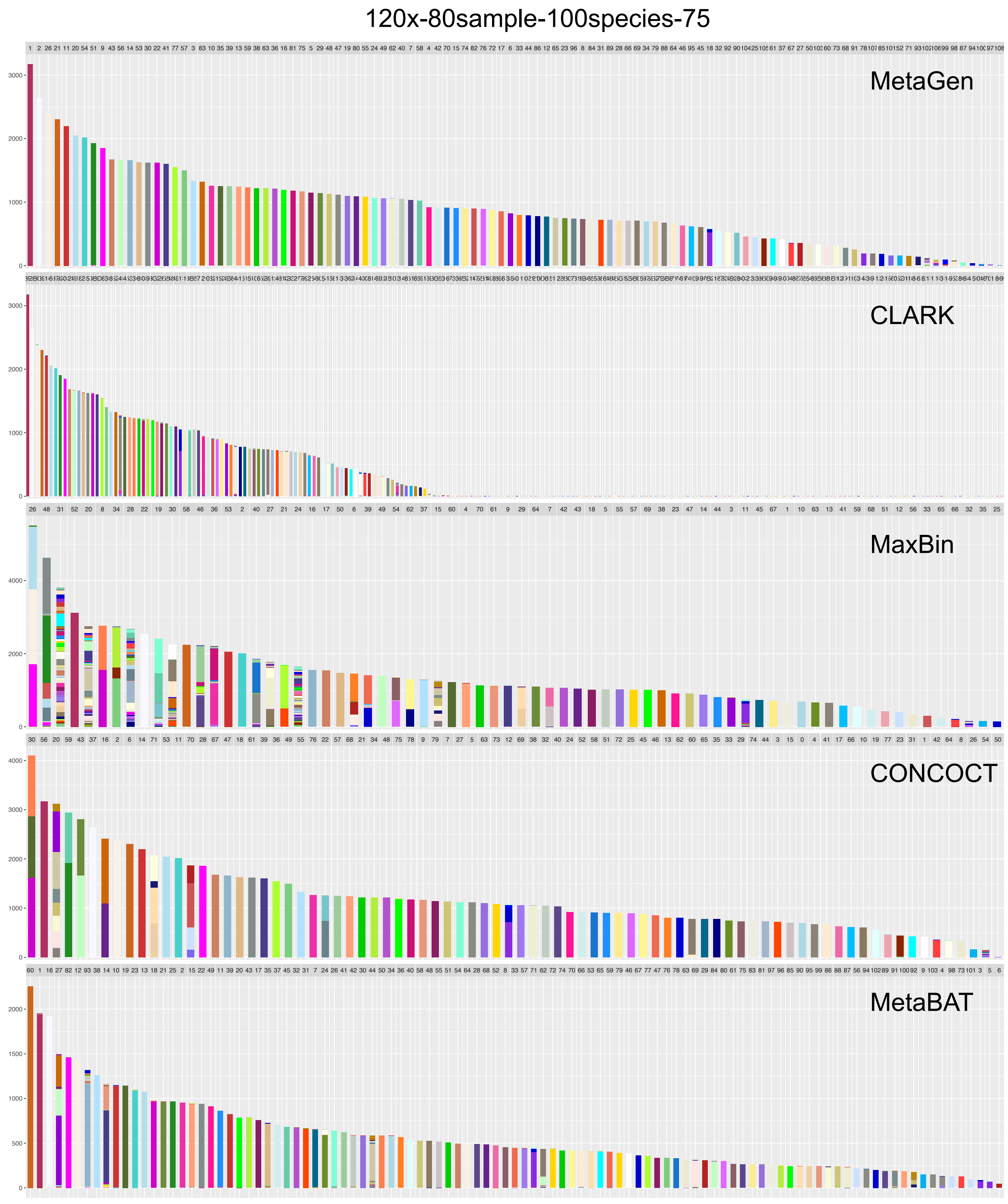

Figure S13: Binning results for MetaGen, CLARK, MaxBin, CONCOCT and MetaBAT with the pooled sequencing depth at 120**x**, the number of samples at 80, the number of species at 150, and with 75% of species in each sample. Color legends are given in **Figure S16**.

Simulation Setting 7:

We fixed the pooled sequencing depth at 120**x**, the number of samples at 80, and the number of specie at 100, and used two different assemblers, MegaHIT and Ray. Performances of MetaGen, CLARK, MaxBin, MetaBAT and CONCOCT are shown in Figure **S14**. The detailed binning results of MetaGen, CLARK, MaxBin, MetaBAT and CONCOCT are shown in Figures **S15, 5**.

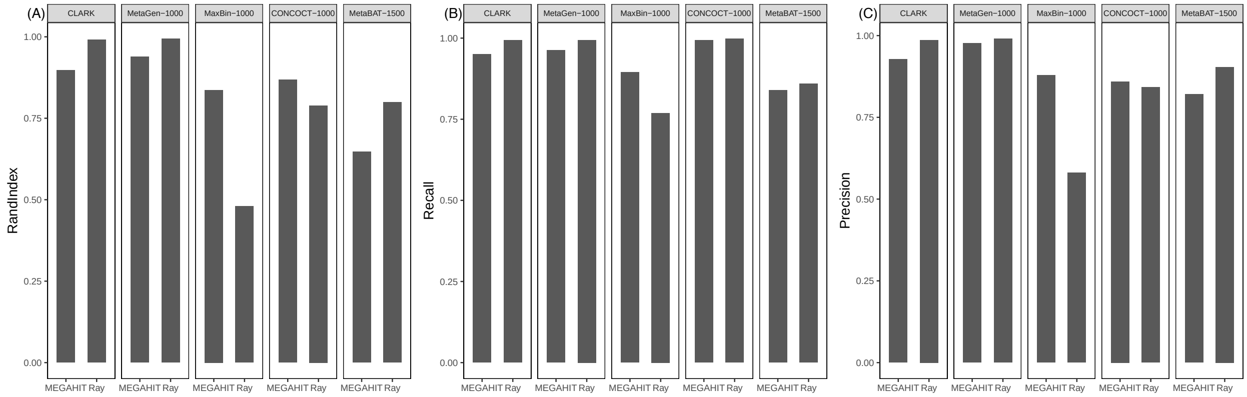

Figure S14: Adjusted Rand Index (A), Precision (B), and Recall (C) of CLARK, MetaGen, MaxBin, CONCOCT, and MetaBAT are evaluated using two different assemblers, MegaHIT and Ray. The simulated data sets have 80 samples, 100 species and the sequencing depth of **120x**.

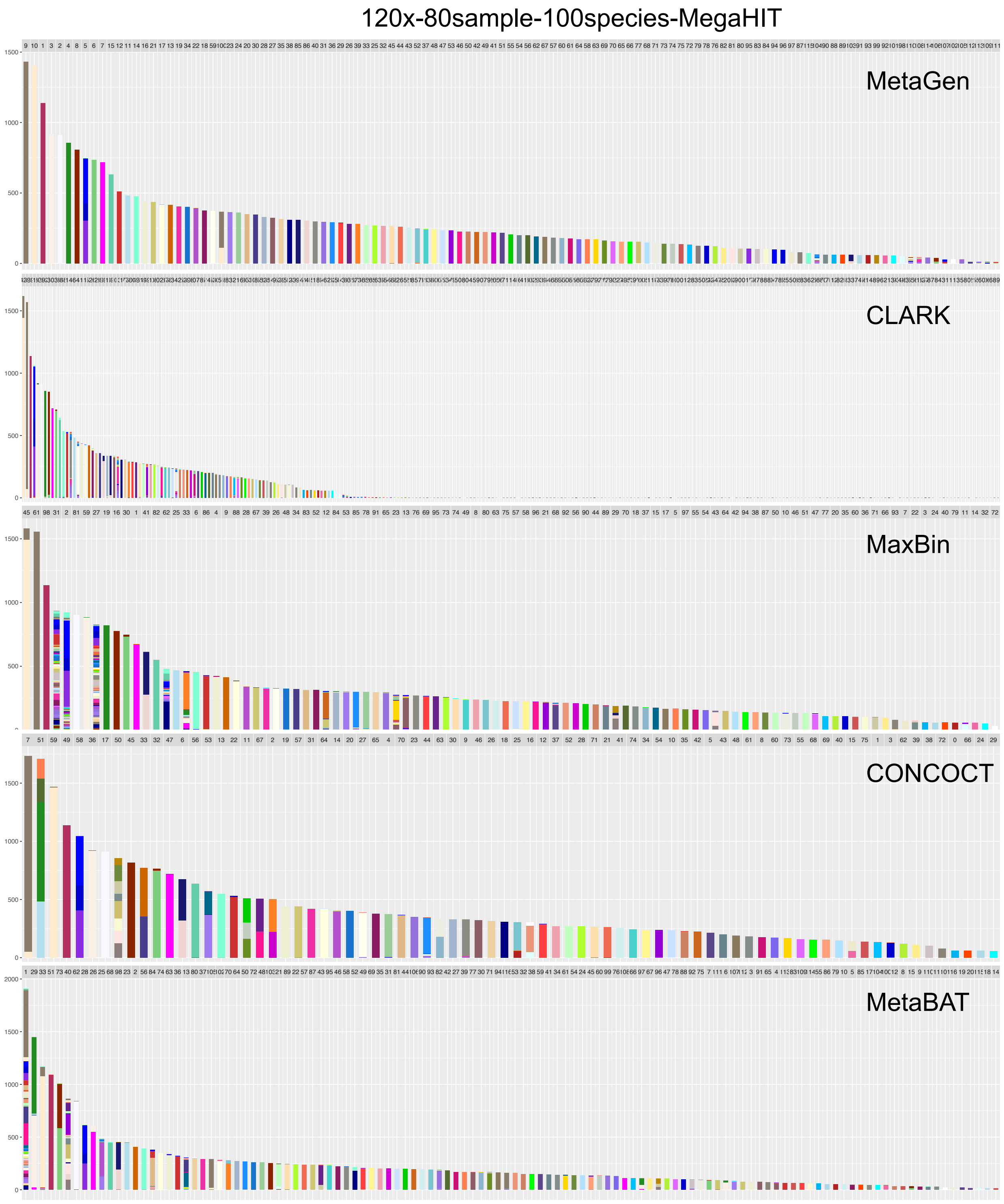

Figure S15: Binning results for MetaGen, CLARK, MaxBin, CONCOCT, and MetaBAT with the pooled sequencing depth at 120**x**, the number of samples at 80, and the number of species at 150, using the MegaHIT assembler. Color legends are given in **Figure S16**.

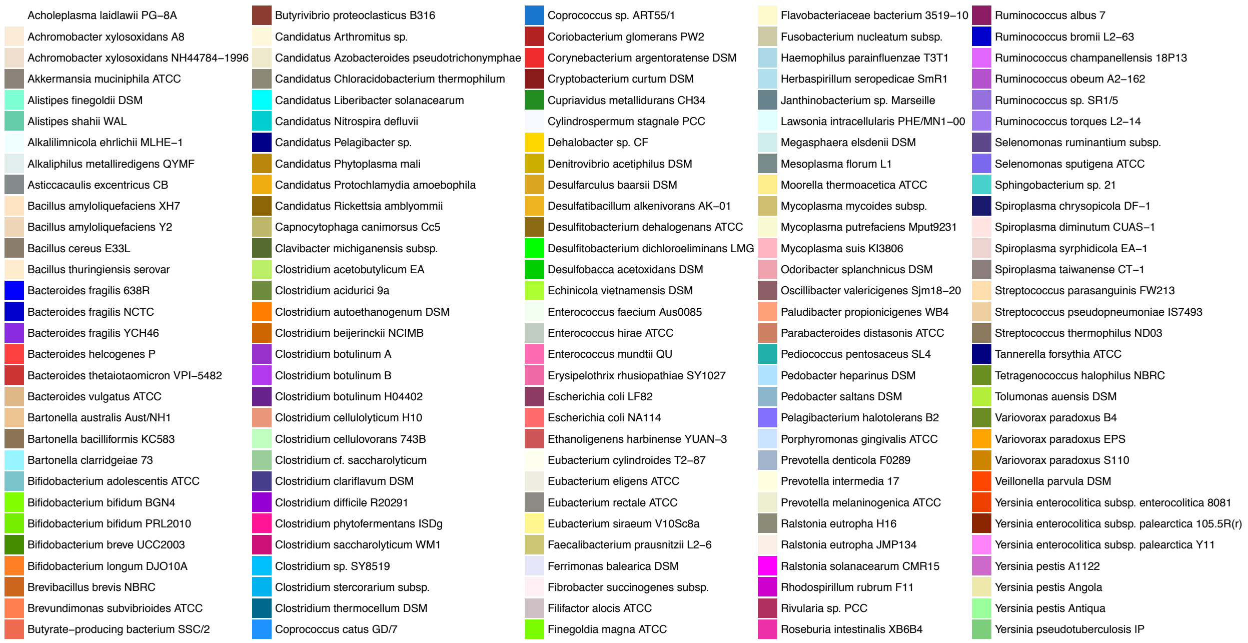

Figure S16: Color codes for all the 150 species used in simulation settings 4-5.

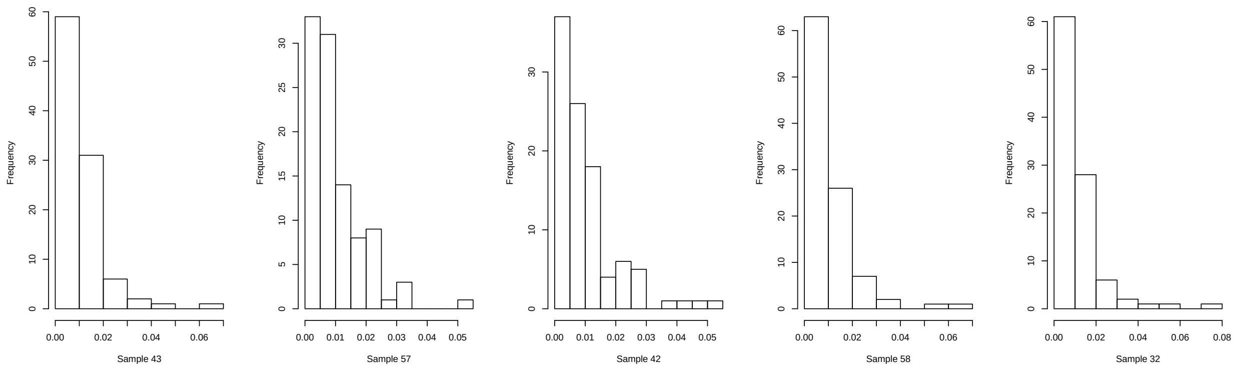

Figure S17: Histogram of the relative abundance of different species for the 5 randomly selected samples for the metagenomic data sets with the pooled sequencing depth at 80**x**, the number of samples at 80, and the number of species at 100.

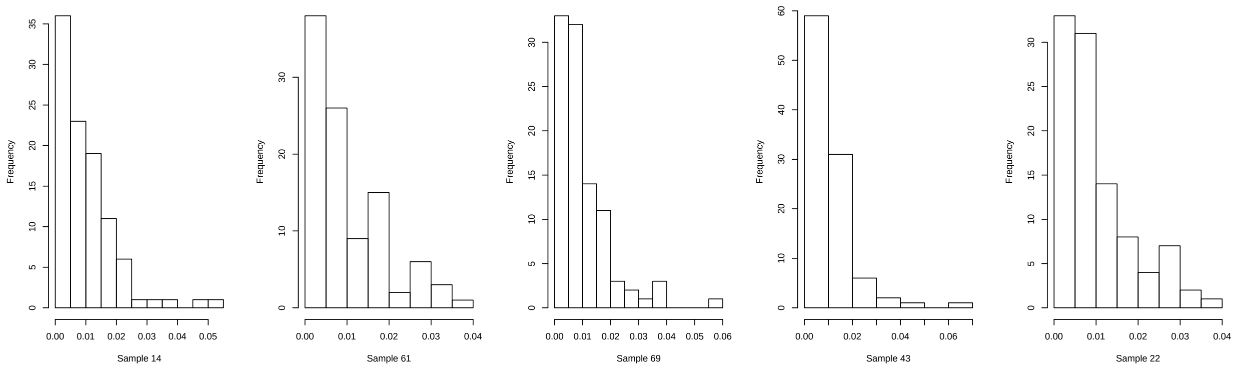

Figure S18: Histogram of the relative abundance of different species for the 5 randomly selected samples for the metagenomic data sets with the pooled sequencing depth at 120**x**, the number of samples at 80, and the number of species at 100.

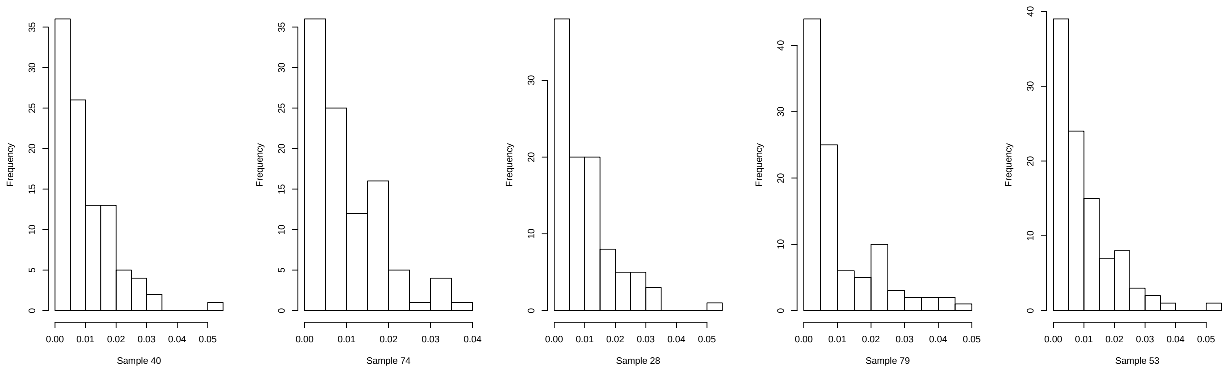

Figure S19: Histogram of the relative abundance of different species for the 5 randomly selected samples for the metagenomic data sets with the pooled sequencing depth at 160**x**, the number of samples at 80, and the number of species at 100.

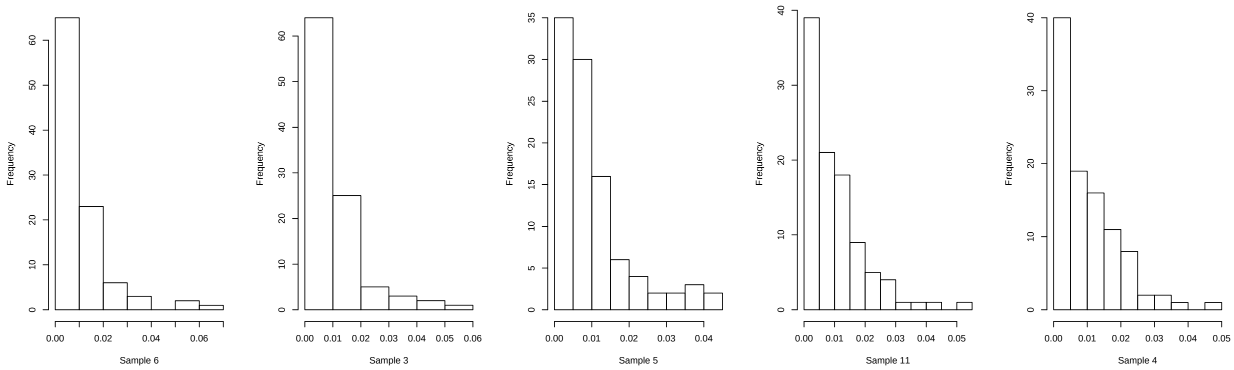

Figure S20: Histogram of the relative abundance of different species for the 5 randomly selected samples for the metagenomic data sets with the pooled sequencing depth at 120**x**, the number of samples at 20, and the number of species at 100.

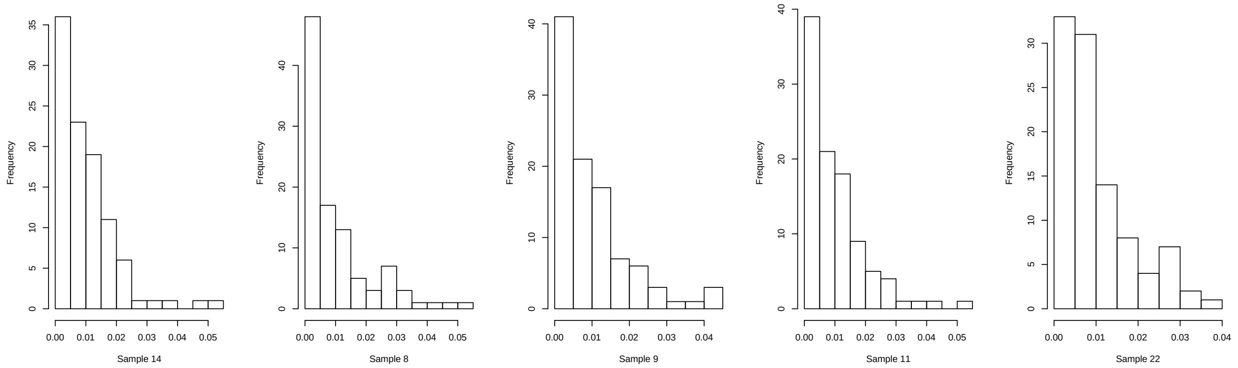

Figure S21: Histogram of the relative abundance of different species for the 5 randomly selected samples for the metagenomic data sets with the pooled sequencing depth at 120**x**, the number of samples at 40, and the number of species at 100.

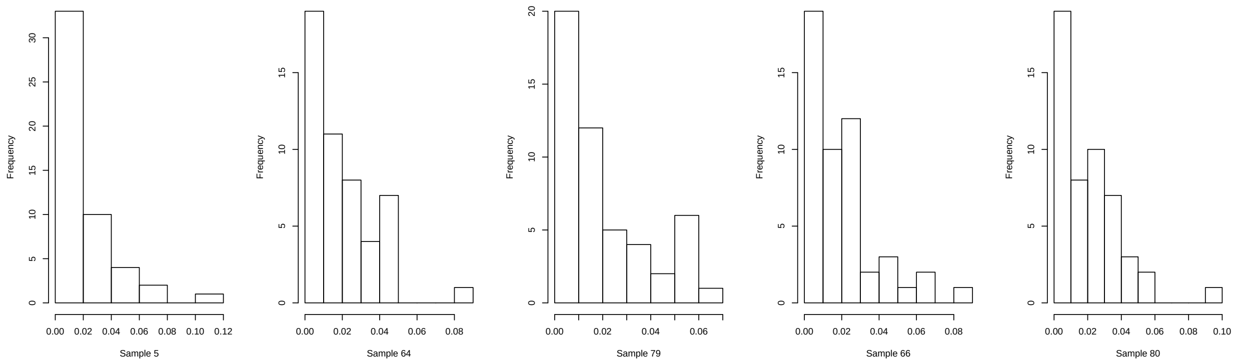

Figure S22: Histogram of the relative abundance of different species for the 5 randomly selected samples for the metagenomic data sets with the pooled sequencing depth at 120**x**, the number of samples at 80, and the number of species at 50.

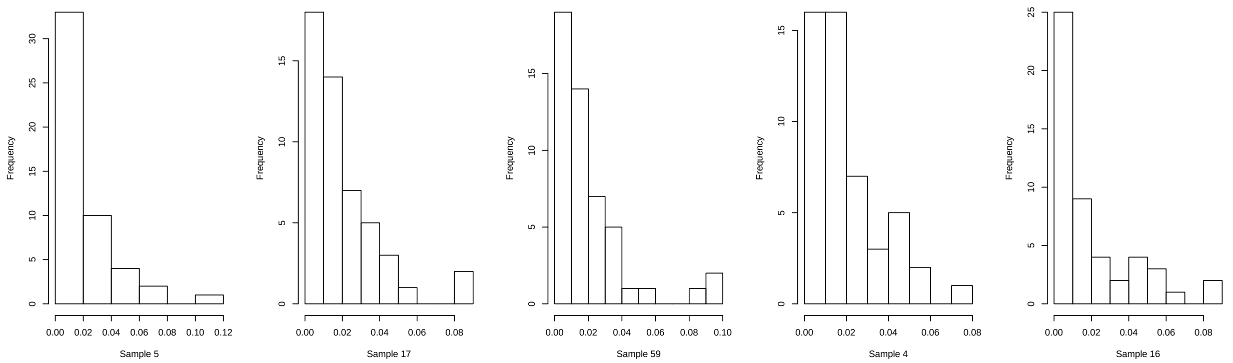

Figure S23: Histogram of the relative abundance of different species for the 5 randomly selected samples for the metagenomic data sets with the pooled sequencing depth at 120**x**, the number of samples at 80, and the number of species at 150.

2 Software parameter settings

We used the default parameter settings for MetaGen, MaxBin and MetaBAT for automatically selecting the number of species. For CONCOCT, we set the true number of species as the maximum number of species and kept the other settings as default. For the reference-based method CLARK, we selected the "bacteria" database and set the taxonomy rank as "species". To map the contigs assembled in our real data examples, we searched the NCBI nucleotide database and used TAXAassign (https://github.com/umerijaz/TAXAassign) to assign them to taxonomic groups. We set the thresholds for the minimum percentage identity as $60, 70, 80, 95, 95, 97$ for phylum, class, order, family, genus, species, respectively.

In the simulation study, we ran MetaGen with default pipline in our software user manual to do the pooled assembly (with Ray or MEGAHIT assembler) and extract the read counting mapping matrix. For simulation 1-4 and 6-7, we use parameter settings -s 10 -i 10 -o 2 (with -t 0.05 for the case with number of sample less or equal than 10). For simulation setting 5, we use the parameter settings -t 0.01 -s 10 -i 10 -o 2. As documented in our user manual, the option -t is used to specify the threshold for the initial values. It is recommended to set this number smaller(0.01 -0.1) when the number of samples is less than 10 and (0.1-0.2) when the number of samples is larger than 10. The default value is set as 0.1. The option -s sets the minimum number of bins. The option -i sets the step size to search the optimal number of species using BIC criterion.

3 Real Data Sets

Metagenomic Analysis of IBD

In this study, we assembled the paired-end reads published in Qin et al. [4] using Ray assembler [5], which generated a total of 3,476,781 contigs. We applied MetaGen on the 71,4582 contigs that are longer than 500bp and identified 2,150 clusters according to our BIC scores (Figure **S24**). We also aligned all the contigs to the NCBI nucleotide database using BLAST, and found that 47,565 contigs have a close match to the species in the database.

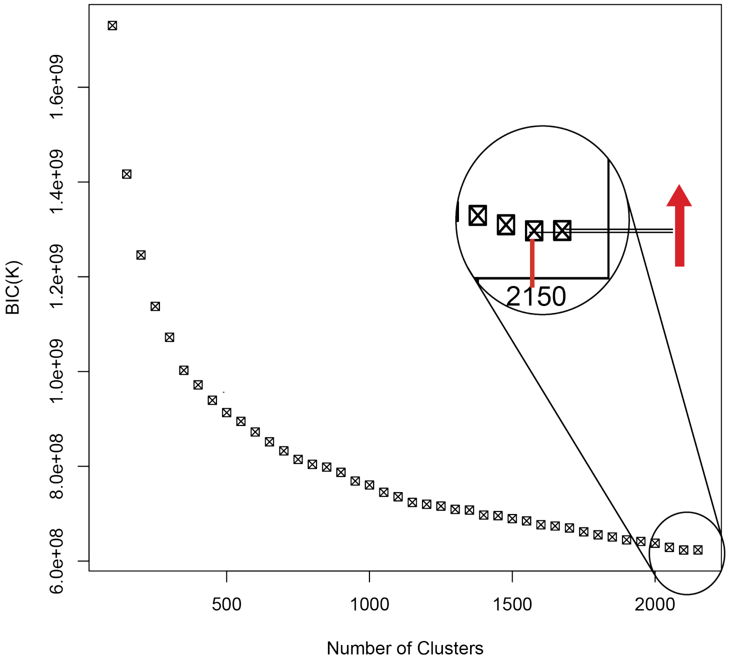

Figure S24: BIC scores of MetaGen for the IBD dataset containing 124 metagenomic samples.

Metagenomic Analysis for T2D

In this study, we assembled the paired-end reads published in Qin et al. [6] using Ray assembler [5], which generated a total of 465, 496 contigs that are longer than 500bp. In Figure **S25**, the BIC score is minimized when the number of clusters reaches 2450. Among all the contigs, 44, 297 can be aligned to reference genomes in the NCBI nucleotide database using BLAST.

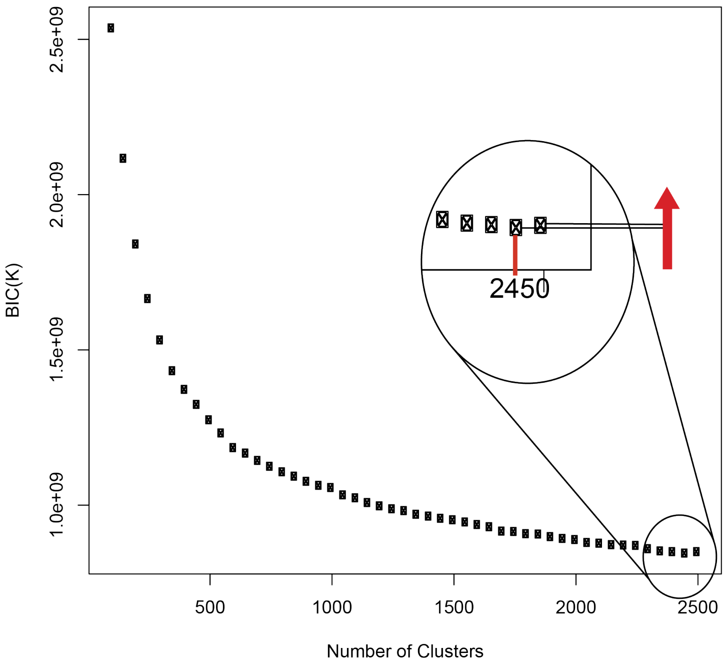

Figure S25: BIC scores of MetaGen for the T2D dataset containing 145 metagenomic samples.

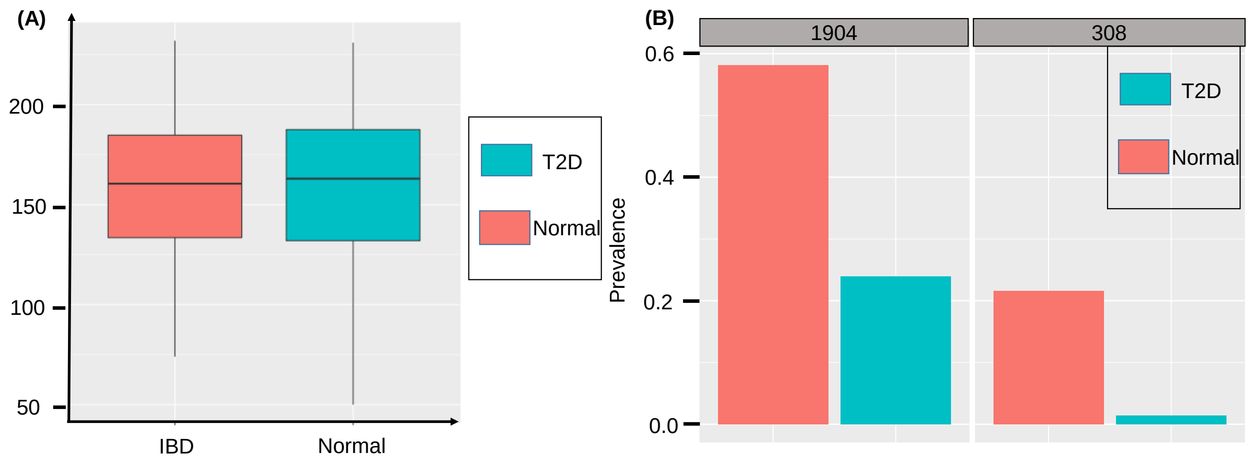

Figure S26: The boxes in (A) represent the distributions of the number of bacterial species in individuals of the normal control and T2D groups, respectively. Plotted in (B) are the prevalences of the 2 clusters (1904 and 308) that are less commonly seen in T2D patients compared to normal controls.

Metagenomic Analysis for Obesity

The DNA samples were sequenced using Pyrosequencing 454 with 9,395,811 total reads for all samples. The pooled short reads from 18 samples were assembled into contigs by the Ray Assembler [5]. A total of 712,751 contigs were constructed, among which 33,107 are longer than 500bp. We further removed 7,724 contigs with fewer than 10 total mapped reads.

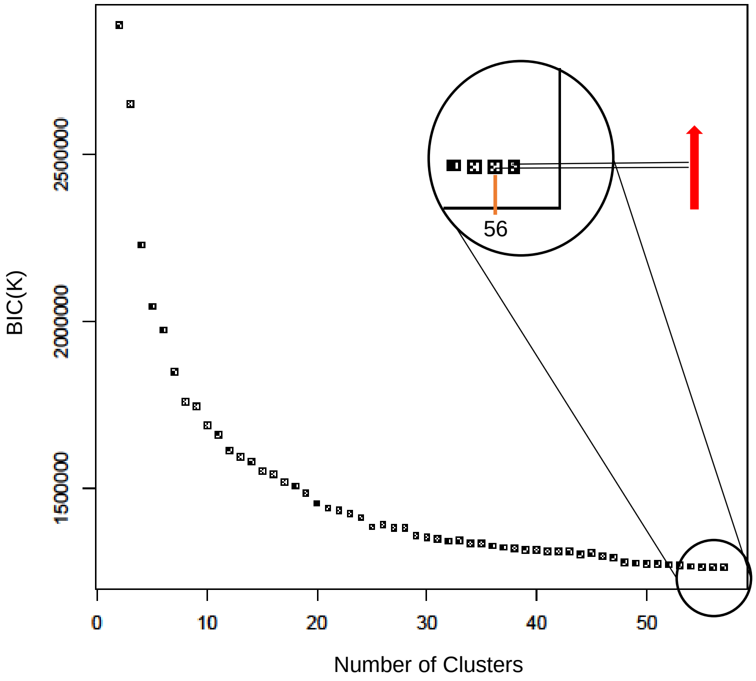

Figure S27: BIC scores of MetaGen for the obesity dataset containing 18 metagenomic samples.

4 Supplementary Note

LASSO-logistic regression for real data

Let y_j denote the group membership of the jth individual, where $y_j = 0$ indicates the control group and $y_j = 1$, the patient group. The logistic regression model assumes that

$$\log \frac{Pr(y_j = 0)}{Pr(y_j = 1)} = \beta_0 + \beta_1 \log(\hat{b}_{j1}) + \cdots + \beta_K \log(\hat{b}_{jK}) \quad (1)$$

where $\hat{b}_{jk}$ is the relatvie species abundance(see **Materials and Methods** (7)). To alleviate the overfitting, we employed LASSO penalty on $\beta_0, \cdots, \beta_K$ by assuming $\sum_{k=1}^{K} |\beta_k| \leq \tau$, where τ is selected by leave-one-out cross validation [7].

Calculating the phylum level relative abundance

Bins are pooled into one phylum if more than 50% of contigs in these bins are mapped to that phylum. For the obesity data set, there are 25 bins that can be mapped to one of the four given phyla. We calculated the relative abundance (see **Materials and Methods**) for each phylum by aggregating the relative abundance of all identified species in this phylum.

Prediction result with CLARK

We applied the same prediction procedure to predict the disease state using the relative abundance outputted by CLARK. The leave-one-out of cross validation error rate is 0.137 and 0.290 for the IBD and T2D data set, which is higher than the error rate predicted by MetaGen.

Supplementary Tables for species name and taxon ID

Species Name	Taxon ID	Species Name	Taxon ID
Acholeplasma laidlawii PG-8A	441768	Achromobacter xylosoxidans A8	762376
Achromobacter xylosoxidans NH44784-1996	1167634	Akkermansia muciniphila ATCC BAA 835	349741
Alistipes finegoldii DSM 17242	679935	Alistipes shahii WAL 8301	717959
Alkalilimnicola ehrlichii MLHE-1	187272	Alkaliphilus metalliredigens QYMF	293826
Asticcacaulis excentricus CB 48	573065	Bacillus amyloliquefaciens XH7	1034836
Bacillus amyloliquefaciens Y2	1126211	Bacillus cereus E33L	288681
Bacillus thuringiensis serovar konkukian 97 27	1279365	Bacteroides fragilis 638R	862962
Bacteroides fragilis NCTC 9343	272559	Bacteroides fragilis YCH46	295405
Bacteroides helcogenes P 36 108	693979	Bacteroides thetaiotaomicron VPI 5482	226186
Bacteroides vulgatus ATCC 8482	435590	Bartonella australis Aust/NH1	1094489
Bartonella bacilliformis KC583	360095	Bartonella clarridgeiae 73	696125
Bifidobacterium adolescentis ATCC 15703	367928	Bifidobacterium bifidum BGN4	484020
Bifidobacterium bifidum PRL2010 chromosome	702459	Bifidobacterium breve UCC2003	326426
Bifidobacterium longum	205913	Brevibacillus brevis NBRC 100599	358681
Brevundimonas subvibrioides ATCC 15264	633149	Butyrate producing bacterium SSC 2	245018
Butyrivibrio proteoclasticus B316	515622	Candidatus Accumulibacter phosphatis clade IIA UW 1	511995
Candidatus Arthromitus sp. SFB-mouse-Yit	1041809	Candidatus Chloracidobacterium thermophilum B	981222
Candidatus Liberibacter solanacearum CLso ZC1	658172	Candidatus Nitrospira defluvii	330214
Candidatus Pelagibacter sp. IMCC9063	1002672	Candidatus Phytoplasma mali	37692
Candidatus Protochlamydia amoebophila UWE25	264201	Candidatus Rickettsia amblyommii str. GAT-30V	1105111
Capnocytophaga canimorsus Cc5	860228	Clavibacter michiganensis sepedonicus	31964
Clostridium acetobutylicum EA 2018	863638	Clostridium acidurici 9a	1128398
Clostridium autoethanogenum DSM 10061	1341692	Clostridium beijerinckii	290402
Clostridium botulinum A str. ATCC 3502	413999	Clostridium botulinum B str. Eklund 17B	935198
Clostridium botulinum H04402 065	941968	Clostridium cellulolyticum H10	394503
Clostridium cellulovorans 743B	573061	Clostridium cf saccharolyticum K10	717608
Clostridium clariflavum DSM 19732	720554	Clostridium difficile R20291	645463
Clostridium phytofermentans ISDg	357809	Clostridium saccharolyticum WM1	610130
Clostridium stercorarium DSM 8532	1121335	Clostridium SY8519	1042156
Clostridium thermocellum DSM 1313	637887	Coprococcus ART55 1	751585

Coprococcus catus GD 7	717962	Coriobacterium glomerans PW2	700015
Corynebacterium argentoratense DSM 44202	1348662	Cryptobacterium curtum DSM 15641	469378
Cupriavidus metallidurans CH34	266264	Cylindrospermum stagnale PCC 7417	56107
Dehalobacter CF	1131462	Denitrovibrio acetiphilus DSM 12809	522772
Desulfarculus baarsii DSM 2075	644282	Desulfatibacillum alkenivorans AK-01	439235
Desulfitobacterium dehalogenans ATCC 51507	756499	Desulfitobacterium dichloroeliminans LMG P 21439	871963
Desulfotomaculum acetoxidans DSM 11109	880072	Echinicola vietnamensis DSM 17526	926556
Enterococcus faecium Aus0085	1305849	Enterococcus hirae ATCC 9790	768486
Enterococcus mundtii QU 25	1300150	Erysipelothrix rhusiopathiae SY1027	1313290
Escherichia coli LF82	591946	Escherichia coli NA114	1033813
Ethanoligenens harbinense YUAN 3	663278	Eubacterium cylindroides T2 87	717960
Eubacterium eligens ATCC 27750	515620	Eubacterium rectale ATCC 33656	515619
Eubacterium siraeum V10Sc8a	717961	Faecalibacterium prausnitzii L2-6	718252
Ferrimonas balearica DSM 9799	550540	Fibrobacter succinogenes subsp. succinogenes S85	59374
Filifactor alocis ATCC 35896	546269	Finegoldia magna ATCC 29328	334413
Flavobacteriaceae bacterium 3519-10	531844	Fusobacterium nucleatum ATCC 25586	190304
Haemophilus parainfluenzae T3T1	862965	Herbaspirillum seropedicae SmR1	757424
Janthinobacterium Marseille	375286	Lawsonia intracellularis PHE MN1 00	363253
Megasphaera elsdenii DSM 20460	1064535	Mesoplasma florum L1	265311
Moorella thermoacetica ATCC 39073	264732	Mycoplasma mycoides capri LC 95010	862259
Mycoplasma putrefaciens Mput9231	1292033	Mycoplasma suis KI3806	708248
Odoribacter splanchnicus DSM 20712	709991	Oscillibacter valericigenes Sjm18-20	693746
Paludibacter propionicigenes WB4	694427	Parabacteroides distasonis ATCC 8503	435591
Pediococcus pentosaceus SL4	1408206	Pedobacter heparinus DSM 2366	485917
Pedobacter saltans DSM 12145	762903	Pelagibacterium halotolerans B2	1082931
Porphyromonas gingivalis ATCC 33277	431947	Prevotella denticola F0289	767031
Prevotella intermedia 17	246198	Prevotella melaninogenica ATCC 25845	553174
Ralstonia eutropha H16	381666	Ralstonia eutropha JMP134	264198
Ralstonia solanacearum CMR15	859655	Rhodospirillum rubrum F11	1036743
Rivularia PCC 7116	373994	Roseburia intestinalis XB6B4	718255
Ruminococcus albus 7	697329	Ruminococcus bromii L2-63	657321
Ruminococcus champanellensis 18P13	213810	Ruminococcus obeum	657314
Ruminococcus sp. SR1/5	657323	Ruminococcus torques L2-14	657313
Selenomonas ruminantium lactilytica TAM6421	927704	Selenomonas sputigena ATCC 35185	546271
Sphingobacterium 21	743722	Spiroplasma chrysopicola DF-1	1276227
Spiroplasma diminutum CUAS 1	1276221	Spiroplasma syrphidicola EA 1	1276229
Spiroplasma taiwanense CT 1	1276220	Streptococcus parasangnis FW213	1114965

Streptococcus pseudopneumoniae IS7493	1054460	Streptococcus thermophilus ND03	767463
Tannerella forsythia ATCC 43037	203275	Tetragenococcus halophilus NBRC 12172	945021
Tolumonas auensis DSM 9187	595494	Variovorax paradoxus B4	1246301
Variovorax paradoxus EPS	595537	Variovorax paradoxus S110	543728
Veillonella parvula DSM 2008	479436	Yersinia enterocolitica palearctica 105 5R r	994476
Yersinia enterocolitica subsp. enterocolitica 8081	393305	Yersinia enterocolitica subsp. palearctica Y11	930944
Yersinia pestis A1122	1035377	Yersinia pestis Angola	349746
Yersinia pestis Antiqua	360102	Yersinia pseudotuberculosis IP 32953	273123

Table 1: 150 genomes used in the simulation study with species names and NCBI taxon id.

Species Name	Taxon ID	Species Name	Taxon ID
E coli 536	362663	E coli APEC O1	405955
E coli IAI1	585034	E coli SE11	409438
E coli DH1	536056	E coli HS	331112

Table 2: 6 *E coli* strains used in the simulation setting 4

Species Name	Taxon ID	Species Name	Taxon ID
Eubacterium rectale	39491	Propionibacterium freudenreichii	1744
Bacteroides vulgatus	821	Conexibacter woesei	191495
Roseburia hominis	301301	Alkalilimnicola ehrlichii	351052
Akkermansia muciniphila	239935	Geobacter uraniireducens	351604
Alistipes finegoldii	214856	Laribacter hongkongensis	168471
Alistipes shahii	328814	Streptococcus anginosus	1328
Eubacterium eligens	39485	Syntrophomonas wolfei	863
Bacteroides thetaiotaomicron	818	Aeromonas hydrophila	644
Odoribacter splanchnicus	28118	Bacillus megaterium	1404
Bacteroides fragilis	817	Corynebacterium aurimucosum	169292
Parabacteroides distasonis	823	Salinibacter ruber	146919
Bacteroides helcogenes	290053	Weissella koreensis	165096
Bifidobacterium adolescentis	1680	Alicycliphilus denitrificans	179636
Prevotella melaninogenica	28132	Rhodospirillum rubrum	1085
Escherichia coli	562	Shigella flexneri	623
Prevotella ruminicola	839	Zobellia galactanivorans	63186
Clostridium sp. SY8519	1042156	Asticcacaulis excentricus	78587
Haemophilus parainfluenzae	729	Herbaspirillum seropedicae	964
Clostridium saccharolyticum	84030	Polaribacter sp. MED152	313598
Oscillibacter valericigenes	351091	Thermoanaerobacterium xy- lanolyticum	29329
Prevotella dentalis	52227	Marivirga tractuosa	1006
Bacteroides salanitronis	376805	Phycisphaera mikurensis	547188
Bifidobacterium longum	216816	Deinococcus proteolyticus	55148
Prevotella intermedia	28131	Geobacter sulfurreducens	35554
Streptococcus suis	1307	Spirochaeta thermophila	154
Prevotella denticola	28129	Streptococcus sp. I-G2	1156431
Prevotella sp. oral taxon 299	652716	Streptococcus intermedius	1338
Ruminococcus champanellensis	1161942	Alkaliphilus metalliredigens	208226
Methanobrevibacter smithii	2173	Brachyspira pilosicoli	52584
Streptococcus thermophilus	1308	Desulfovibrio salexigens	880
Adlercreutzia equolifaciens	446660	Synechococcus sp. WH 7803	32051
Ethanoligenens harbinense	253239	Advenella kashmirensis	310575
Bifidobacterium bifidum	1681	Sinorhizobium medicae	110321
Butyrivibrio proteoclasticus	43305	Streptococcus oralis	1303
Ruminococcus albus	1264	Acetohalobium arabaticum	28187
Enterococcus faecium	1352	Anaeromyxobacter sp. Fw109-5	404589
Eubacterium limosum	1736	Cupriavidus taiwanensis	164546
Lactobacillus delbrueckii	1584	Micavibrio aeruginosavorus	349221
Streptococcus salivarius	1304	Streptococcus agalactiae	1311

Streptococcus lutetiensis	150055	Corynebacterium variabile	1727
Tannerella forsythia	28112	Xanthobacter autotrophicus	280
Gordonibacter pamelaeae	471189	Phenylobacterium zucineum	284016
Veillonella parvula	29466	Ramlibacter tataouinensis	94132
Bifidobacterium breve	1685	Paenibacillus sp. JDR-2	324057
Desulfovibrio desulfuricans	876	Spirochaeta africana	46355
Campylobacter jejuni	197	Thermosediminibacter oceani	291990
Eggerthella lenta	84112	Pseudomonas mendocina	300
Streptococcus parasanguinis	1318	Caldicellulosiruptor kronotskyensis	413889
Clostridium botulinum	1491	Deinococcus maricopensis	309887
Desulfovibrio vulgaris	881	Pseudomonas protegens	380021
Acidaminococcus fermentans	905	Anaeromyxobacter dehalogenans	161493
Enterococcus faecalis	1351	Rhizobium sp. IRBG74	424182
Acidaminococcus intestini	187327	Thermanaerovibrio acidaminovorans	81462
Porphyromonas gingivalis	837	Pseudoxanthomonas suwonensis	314722
Haemophilus influenzae	727	Verminephrobacter eiseniae	364317
Treponema succinifaciens	167	Syntrophobacter fumaroxidans	119484
Eggerthella sp. YY7918	502558	Treponema azotonutricium	150829
Streptococcus pyogenes	1314	Treponema brennaborense	81028
Lactobacillus ruminis	1623	Beijerinckia indica	533
Slackia heliotrinireducens	84110	Acholeplasma brassicae	61635
Olsenella uli	133926	Actinobacillus pleuropneumoniae	715
Lactococcus lactis	1358	Oligotropha carboxidovorans	40137
Salmonella enterica	28901	Wolinella succinogenes	844
Streptococcus pasteurianus	197614	Desulfitobacterium dehalogenans	36854
Porphyromonas asaccharolytica	28123	Rhodococcus equi	43767
Solitalea canadensis	995	Arthrobacter sp. FB24	290399
Bifidobacterium animalis	28025	Magnetococcus marinus	1124597
Selenomonas ruminantium	971	[Bacillus] selenitireducens	85683
Coriobacterium glomerans	33871	Chromohalobacter salexigens	158080
Megasphaera elsdenii	907	Desulfosporosinus acidiphilus	885581
Sorangium cellulosum	56	Methylocystis sp. SC2	187303
Selenomonas sputigena	69823	Corynebacterium glutamicum	1718
Treponema denticola	158	Acidovorax sp. KKS102	358220
Paenibacillus mucilaginosus	61624	Chlorobium limicola	1092
Heliobacterium modesticaldum	35701	Rhizobium etli	29449
Paludibacter propionicigenes	185300	Xanthomonas campestris	339
Bifidobacterium dentium	1689	Brachybacterium faecium	43669
Saprospira grandis	1008	Gloeobacter violaceus	33072
Riemerella anatipestifer	34085	Kocuria rhizophila	72000
Lawsonia intracellularis	29546	Acholeplasma palmae	38986
Streptococcus mitis	28037	Leifsonia xyli	1575

Desulfovibrio gigas	879
Syntrophobotulus glycolicus	51197
Gardnerella vaginalis	2702
Desulfotomaculum acetoxidans	58138
Anaerococcus prevotii	33034
Symbiobacterium thermophilum	2734
Chitinophaga pinensis	79329
Clostridium tetani	1513
Mycobacterium tuberculosis	1773
Clostridium cellulovorans	1493
Pseudomonas stutzeri	316
Clostridium perfringens	1502
Candidatus Azobacteroides pseudotrichonymphae	511435
Clostridium stercorarium	1510
Filifactor alocis	143361
Cytophaga hutchinsonii	985
Clostridium kluyveri	1534
Desulfovibrio magneticus	184917
Clostridium saccharobutylicum	169679
Clostridium clariflavum	288965
Aggregatibacter aphrophilus	732
Rhodopseudomonas palustris	1076
Desulfarculus baarsii	453230
Fusobacterium nucleatum	851
Pseudomonas putida	303
Fibrella aestuarina	651143
Mycoplasma hyopneumoniae	2099
Clostridium saccharoperbutylacetonicum	36745
Finegoldia magna	1260
Flavobacterium johnsoniae	986
Desulfomicrobium baculatum	899
Bifidobacterium thermophilum	33905
Clostridium sp. BNL1100	755731
Brevibacillus brevis	1393
Thermaerobacter marianensis	73919
Desulfotomaculum gibsoniae	102134
Clostridium cellulolyticum	1521
Niastella koreensis	354356
Halothiobacillus neapolitanus	927
Stackebrandtia nassauensis	283811
Thermus thermophilus	274
Actinoplanes missouriensis	1866
Streptobacillus moniliformis	34105
Dichelobacter nodosus	870
Pseudomonas syringae	317
Corynebacterium halotolerans	225326
Klebsiella pneumoniae	573
Macrococcus caseolyticus	69966
Streptococcus pseudopneumoniae	257758
Thermacetogenium phaeum	85874
Tistrella mobilis	171437
Wolbachia sp. wRi	66084
Calditerrivibrio nitroreducens	477976
Candidatus Puniceispirillum marinum	767892
Frateuria aurantia	81475
Micrococcus luteus	1270
Neisseria meningitidis	487
Azorhizobium caulinodans	7
Azotobacter vinelandii	354
Belliella baltica	232259
Marinithermus hydrothermalis	186192
Paenibacillus terrae	159743
Thiobacillus denitrificans	36861
Candidatus Arthromitus sp. SFB-mouse	49118
Myxococcus stipitatus	83455
Pseudovibrio sp. FO-BEG1	911045
Candidatus Accumulibacter phosphatis	327160
Catenulispora acidiphila	304895
Geobacter bemidjiensis	225194
Oceanimonas sp. GK1	511062
Spiroplasma taiwanense	2145
Elusimicrobium minutum	423605
Novosphingobium sp. PP1Y	702113
Clavibacter michiganensis	28447
Leptotrichia buccalis	40542
Myxococcus xanthus	34
Saccharothrix espanaensis	103731
Flavobacterium branchiophilum	55197

Clostridium acetobutylicum	1488	Gemmatimonas aurantiaca	173480
Thermobacillus composti	377615	Polymorphum gilvum	991904
Acetobacterium woodii	33952	Bacillus cereus	1396
Desulfovibrio africanus	873	Caldilinea aerophila	133453
Capnocytophaga ochracea	1018	Cyanothece sp. ATCC 51142	43989
Pyrolobus fumarii	54252	Ruegeria pomeroyi	89184
Fibrobacter succinogenes	833	Arthrospira platensis	118562
Mahella australiensis	252966	Desulfohalobium retbaense	45663
Pedobacter heparinus	984	Geobacillus sp. C56-T3	691437
Aeromonas salmonicida	645	Myxococcus fulvus	33
Desulfurispirillum indicum	936456	Azoarcus sp. KH32C	748247
Dyadobacter fermentans	94254	Caldisericum exile	693075
Lactobacillus acidophilus	1579	Azospirillum brasilense	192
Melioribacter roseus	1134405	Brevundimonas subvibrioides	74313
Dehalococcoides mccartyi	61435	Desulfosporosinus orientis	1563
Enterobacter cloacae	550	Marinobacter hydrocarbonoclasticus	2743
Pseudomonas aeruginosa	287	Streptomyces cattleya	29303
Serratia marcescens	615	Agrobacterium fabrum	1176649
Achromobacter xylosoxidans	85698	Polynucleobacter necessarius	576610
Azospirillum lipoferum	193	Thermobifida fusca	2021
Clostridium novyi	1542	Burkholderia pseudomallei	28450
Desulfitobacterium hafniense	49338	Cupriavidus metallidurans	119219
Desulfotomaculum ruminis	1564	Ferrimonas balearica	44012
Streptococcus pneumoniae	1313	Cellvibrio japonicus	155077
Bacillus coagulans	1398	Sideroxydans lithotrophicus	63745
Desulfovibrio aespoeensis	182210	Burkholderia sp. YI23	1097668
Rhizobium leguminosarum	384	Deinococcus peraridilitoris	432329
Ralstonia solanacearum	305	Geitlerinema sp. PCC 7407	1173025
Haliangium ochraceum	80816	Mycoplasma agalactiae	2110
Desulfitobacterium dichloroeliminans	233055	Caldicellulosiruptor saccharolyticus	44001
Escherichia fergusonii	564	Mycobacterium abscessus	36809
Variovorax paradoxus	34073	Streptomyces bingchenggensis	379067
Cyanobium gracile	59930	Candidatus Amoebophilus asiaticus	281120
Atopobium parvulum	1382	Carnobacterium maltaromaticum	2751
Erysipelothrix rhusiopathiae	1648	Paracoccus denitrificans	266
Chromobacterium violaceum	536	Sphingomonas sp. MM-1	745310
Sphaerobacter thermophilus	2057	Zymomonas mobilis	542
Desulfovibrio alaskensis	58180	Chamaesiphon minutus	1173032
Alicyclobacillus acidocaldarius	405212	Pelobacter carbinolicus	19
Clostridium beijerinckii	1520	Sebaldella termitidis	826
Staphylococcus aureus	1280	Singulisphaera acidiphila	466153
Geobacter sp. M21	443144	Sphingobium sp. SYK-6	627192

Rhodothermus marinus	29549	Burkholderia thailandensis	57975
Sphingobacterium sp. 21	743722	Gluconacetobacter diazotrophicus	33996
Mycoplasma pulmonis	2107	Kytococcus sedentarius	1276
Clostridium pasteurianum	1501	Nitratifractor salsuginis	269261
Rubrivivax gelatinosus	28068	Staphylococcus saprophyticus	29385
Desulfatibacillum alkenivorans	259354	Synechococcus sp. JA-3-3Ab	321327
Francisella tularensis	263	Arthrobacter sp. Rue61a	1118963
Aromatoleum aromaticum	551760	Coprothermobacter proteolyticus	35786
Mobiluncus curtisii	2051	Flavobacteriaceae bacterium 3519-10	531844
Rhodospirillum centenum	34018	Shewanella amazonensis	60478
Desulfobacterium autotrophicum	2296	Syntrophus aciditrophicus	316277
Desulfobulbus propionicus	894	Thermoanaerobacter wiegelii	46354
Candidatus Desulforudis audaxviator	471827	Deinococcus radiodurans	1299
Candidatus Solibacter usitatus	332163	Rhizobium tropici	398
Runella slithyformis	106	Yersinia enterocolitica	630
Geobacter metallireducens	28232	Candidatus Koribacter versatilis	658062
Alkaliphilus oremlandii	461876	Corynebacterium maris	575200
Leptothrix cholodnii	34029	Hyphomicrobium denitrificans	53399
Moorella thermoacetica	1525	Streptomyces coelicolor	1902
Pseudogulbenkiania sp. NH8B	748280	Bacillus infantis	324767
Beutenbergia cavernae	84757	Desulfomonile tiedjei	2358
Owenweeksia hongkongensis	253245	Granulicella tundricola	940615
Pediococcus pentosaceus	1255	Ilyobacter polytropus	167642
Carboxydothermus hydrogenoformans	129958	Rhodobacter capsulatus	1061
Paenibacillus sp. Y412MC10	481743	Bacillus clausii	79880
Tepidanaerobacter acetatoxydans	499229	Gallionella capsiferriformans	370405
Stenotrophomonas maltophilia	40324	Pectobacterium carotovorum	554
Comamonas testosteroni	285	Aequorivita sublithincola	101385
Robiginitalea biformata	252307	Anaerolinea thermophila	167964
Treponema primitia	88058	Halanaerobium praevalens	2331
Paenibacillus larvae	1464	Sphaerochaeta pleomorpha	1131707
Geobacter sp. M18	443143	Erwinia billingiae	182337
Thiomicrospira crunogena	39765	Pelagibacterium halotolerans	531813
Campylobacter coli	195	Anoxybacillus flavithermus	33934
Ignavibacterium album	591197	Collimonas fungivorans	158899
Pelobacter propionicus	29543	Desulfovibrio piezophilus	879567
Thermoanaerobacterium thermosaccharolyticum	1517	Methylobacterium sp. 4-46	426117
Gluconobacter oxydans	442	Rothia dentocariosa	2047
Acholeplasma laidlawii	2148	Sulfuricella denitrificans	649841
Streptococcus sanguinis	1305	Thermomonospora curvata	2020
Streptomyces violaceusniger	68280	Bdellovibrio bacteriovorus	959

Magnetospirillum gryphiswaldense	55518	Burkholderia mallei	13373
Lactobacillus reuteri	1598	Frankia alni	1859
Allochromatium vinosum	1049	Methylomonas methanica	421
Jonesia denitrificans	43674	Pasteurella multocida	747
Thauera sp. MZ1T	85643	Prosthecochloris aestuarii	1102
Bacillus cellulosilyticus	1413	Starkeya novella	921
Magnetospirillum magneticum	84159	Terriglobus roseus	392734
Stigmatella aurantiaca	41	Thermus scotoductus	37636
Flavobacterium columnare	996	Chloracidobacterium thermophilum	458033
Methylomicrobium alcaliphilum	271065	Chlorobium phaeovibrioides	1094
Pseudomonas fluorescens	294	Corynebacterium efficiens	152794
Bifidobacterium asteroides	1684	Geobacter daltonii	1203471
Chlorobaculum parvum	274539	Nakamurella multipartita	53461
Geobacter lovleyi	313985	Streptococcus constellatus	76860
Listeria monocytogenes	1639	Ammonifex degensii	42838
Mycoplasma hyorhinis	2100	Polaromonas sp. JS666	296591
Cupriavidus necator	106590	Rhodococcus erythropolis	1833
Rothia mucilaginosa	43675	Amphibacillus xylanus	1449
Paenibacillus polymyxa	1406	Halobacillus halophilus	1570
Streptococcus gordonii	1302	Methylococcus capsulatus	414
Lactobacillus salivarius	1624	Mycoplasma penetrans	28227
Zunongwangia profunda	398743	Photorhabdus asymbiotica	291112
Streptococcus equi	1336	Hirschia baltica	2724
Thermincola potens	863643	Ochrobactrum anthropi	529
Buchnera aphidicola	9	Blastococcus saxobsidens	138336
Cellulophaga lytica	979	Cryptobacterium curtum	84163
Desulfotomaculum reducens	59610	Natranaerobius thermophilus	375929
Streptosporangium roseum	2001	Nocardia cyriacigeorgica	135487
Corallococcus coralloides	184914	Thiomonas intermedia	926
Solibacillus silvestris	76853	Burkholderia sp. RPE64	758793
Desulfurivibrio alkaliphilus	427923	Deinococcus deserti	310783
Gallibacterium anatis	750	Kineococcus radiotolerans	131568
Ralstonia pickettii	329	Mesorhizobium ciceri	39645
Shigella sonnei	624	Vibrio furnissii	29494
Streptococcus sp. I-P16	1156433	Bacillus cytotoxicus	580165
Thiocystis violascens	73141	Deinococcus geothermalis	68909
Citrobacter rodentium	67825	Pantoea sp. At-9b	592316
Prochlorococcus marinus	1219	Sulfurimonas autotrophica	202747
Weeksella virosa	1014	Corynebacterium jeikeium	38289
Shigella boydii	621	Nitrospira defluvii	330214
Deinococcus gobiensis	502394	Echinicola vietnamensis	390884
Haliscomenobacter hydrossis	2350	Lactococcus garvieae	1363

Leadbetterella byssophila	316068	Nocardiopsis dassonvillei	2014
Oceanobacillus iheyensis	182710	Turneriella parva	29510
Parvibaculum lavamentivorans	256618	Amycolatopsis mediterranei	33910
Desulfococcus oleovorans	181663	Bacillus sp. 1NLA3E	666686
Hyphomonas neptunium	81032	Pseudomonas resinovorans	53412
Microlunatus phosphovorus	29405	Pusillimonas sp. T7-7	1007105
Lactobacillus fermentum	1613	Thermus sp. CCB_US3_UF1	1111069
Oceanithermus profundus	187137	Candidatus Phytoplasma australiense	59748
Rhodobacter sphaeroides	1063	Edwardsiella ictaluri	67780
Rubrobacter xylanophilus	49319	Methylibium petroleiphilum	105560
Staphylococcus haemolyticus	1283	Xenorhabdus bovienii	40576
Thioflavicoccus mobilis	80679	Caulobacter segnis	88688
Acetobacter pasteurianus	438	Novosphingobium aromaticivorans	48935
Bacillus subtilis	1423	Ornithobacterium rhinotracheale	28251
Agrobacterium sp. H13-3	861208	Polaromonas naphthalenivorans	216465
Bacillus thuringiensis	1428	Roseiflexus sp. RS-1	357808
Bordetella petrii	94624	Truepera radiovictrix	332249
Nautilia profundicola	244787	Bordetella avium	521
Opitutus terrae	107709	Burkholderia gladioli	28095
Sinorhizobium fredii	380	Erwinia amylovora	552
Meiothermus ruber	277	Exiguobacterium sibiricum	332410
Shigella dysenteriae	622	Frankia sp. EAN1pec	298653
Acidobacterium capsulatum	33075	Melissococcus plutonius	33970
Brachyspira hyodysenteriae	159	Thermobispora bispora	2006
Dechloromonas aromatica	259537	Methylobacterium nodulans	114616
Gordonia sp. KTR9	337191	Sphingomonas wittichii	160791
Methylobacterium extorquens	408	Rhodomicrobium vannielii	1069
Rivularia sp. PCC 7116	373994		

Table 3: 545 species used in the simulation setting 5

References

[1] Daniel C Richter, Felix Ott, Alexander F Auch, Ramona Schmid, and Daniel H Huson. Metasim—a sequencing simulator for genomics and metagenomics. *PloS One*, 3(10):e3373, 2008.

[2] Weichun Huang, Leping Li, Jason R Myers, and Gabor T Marth. Art: a next-generation sequencing read simulator. *Bioinformatics*, 28(4):593–594, 2012.

[3] Chengwei Luo, Rob Knight, Heli Siljander, Mikael Knip, Ramnik J Xavier, and Dirk Gevers.

Constrains identifies microbial strains in metagenomic datasets. *Nature biotechnology*, 33(10): 1045–1052, 2015.

[4] Junjie Qin, Ruiqiang Li, Jeroen Raes, Manimozhiyan Arumugam, Kristoffer Solvsten Burgdorf, Chaysavanh Manichanh, Trine Nielsen, Nicolas Pons, Florence Levenez, Takuji Yamada, et al. A human gut microbial gene catalogue established by metagenomic sequencing. *nature*, 464 (7285):59–65, 2010.

[5] Sébastien Boisvert, Frédéric Raymond, Élénie Godzaridis, François Laviolette, Jacques Corbeil, et al. Ray meta: scalable de novo metagenome assembly and profiling. *Genome Biol*, 13(12): R122, 2012.

[6] Junjie Qin, Yingrui Li, Zhiming Cai, Shenghui Li, Jianfeng Zhu, Fan Zhang, Suisha Liang, Wenwei Zhang, Yuanlin Guan, Dongqian Shen, et al. A metagenome-wide association study of gut microbiota in type 2 diabetes. *Nature*, 490(7418):55–60, 2012.

[7] Robert Tibshirani. Regression shrinkage and selection via the lasso. *Journal of the Royal Statistical Society. Series B (Methodological)*, pages 267–288, 1996.
